# Accurate stock movement prediction via centroid-based randomness smoothing

**DOI:** 10.1371/journal.pone.0345252

**Published:** 2026-10-08

**Authors:** Yejun Soun, Hosung Lee, U Kang

**Affiliations:** Department of Computer Science and Engineering, Seoul National University, Seoul, Republic of Korea; BRAC University, BANGLADESH

## Abstract

How can we identify stable patterns to accurately predict stock price movements amidst severe market noise? Predicting stock price movements is a crucial problem in financial data mining, and has attracted significant attention from researchers and financial institutions. Although there have been several works on learning patterns from quantitative data to predict stock prices, they encounter critical challenges from data uncertainty and model limitations. Data uncertainty arises in financial data because asset prices are determined by the market. Different participants hold distinct valuations for the same asset, which causes prices to fluctuate. This inherent randomness makes it difficult to identify consistent patterns in the data. Additionally, because of this inherent randomness, existing stock movement prediction models that repeatedly stack more recurrent layers end up propagating the randomness forward. It thus becomes more difficult to capture the complex patterns of stock prices. In this work, we propose Craft (Centroid-based Randomness Smoothing Approach for Stock Forecasting with Transformer Architecture), an accurate stock movement prediction model designed to extract clear patterns in stock data and perform refined forecasting through a Transformer-based architecture. By applying an effective randomness smoothing process, Craft uncovers meaningful and consistent patterns that facilitate accurate predictions. We evaluate Craft on fourteen test settings spanning six real-world datasets and three market regimes. Craft achieves the highest prediction accuracy in twelve of these settings and ranks the second in the other two. Craft also improves the annualized Sharpe ratio by up to 1.0 while reducing the relative maximum drawdown by up to 7.0% points over the best competitor.

## Introduction

Given price data with inherent randomness, how can we identify consistent patterns and make precise predictions of price movements? Stock movement prediction is one of the fields with the smallest gap between fundamental research and economic benefits, making it a topic of great interest to many researchers and financial institutions. Accurately predicting stock movements has a significant impact on financial investments.

However, stock movement prediction entails challenges commensurate with its economic utility due to the following three main limitations. First, stock prices exhibit fluctuations and a significant level of unexplainable fluctuations due to the diverse range of entities involved in trading. Second, the movement of an individual stock is difficult to explain solely by its own dynamics due to the influence of numerous external variables beyond the company’s inherent factors [1,2]. Third, recent multi-layer time-series approaches can intensify the inherent randomness in stock data by propagating noise through increasingly complex hidden representations, exacerbating uncertainty in the final predictions.

The main limitation of existing stock prediction methods lies in their excessive emphasis on explaining the inherent randomness of stock price data. They often incorporate external variables such as technical indicators [3,4], fundamental factors [5,6], or macroeconomic data [7]—leading to potential overfitting when the feature space grows faster than the available training instances. Others adopt deeper Transformer-based architectures on raw price features [8,9], but random spikes frequently overshadow the model’s capacity to isolate meaningful patterns. When multiple self-attention layers are stacked in the presence of significant data uncertainty, the resulting network can amplify noise rather than filtering it out, making it difficult to distinguish true signals from volatile fluctuations. Although Transformer-based approaches have shown promise in capturing both local and global dependencies, they typically rely on unprocessed raw price inputs. Thus, the deeper these models go, the more likely they propagate spurious variations, ultimately limiting their effectiveness in highly stochastic financial settings.

In this work, we propose Craft (Centroid-based Randomness Smoothing Approach for Stock Forecasting with Transformer Architecture), an accurate stock movement prediction model designed to extract generalized patterns from the randomness of stock movements (see Fig 1). Craft reduces randomness and identifies clear patterns from stock data with the following three main ideas. First, Craft reduces stock-specific randomness in price data by applying centroid-based clustering methods to stock return data and using the centroids of tokens as token embedding. Second, Craft forms a “glocal” (global + local) context by combining each stock’s local return sequence with a global context built from stock-wise correlations at each time step. The model aggregates local patterns of highly correlated stocks by a weighted average and applies time-axis self-attention for precise next-token predictions. Third, Craft leverages the time-axis glocal representation within an end-to-end framework by adding a stock-axis attention module. This dual-axis design not only retains the local next-token patterns, but also captures asymmetric and dynamic cross-stock correlations, thus enabling Craft to accurately predict both the next token and the next price movement for each stock.

**Fig 1:**
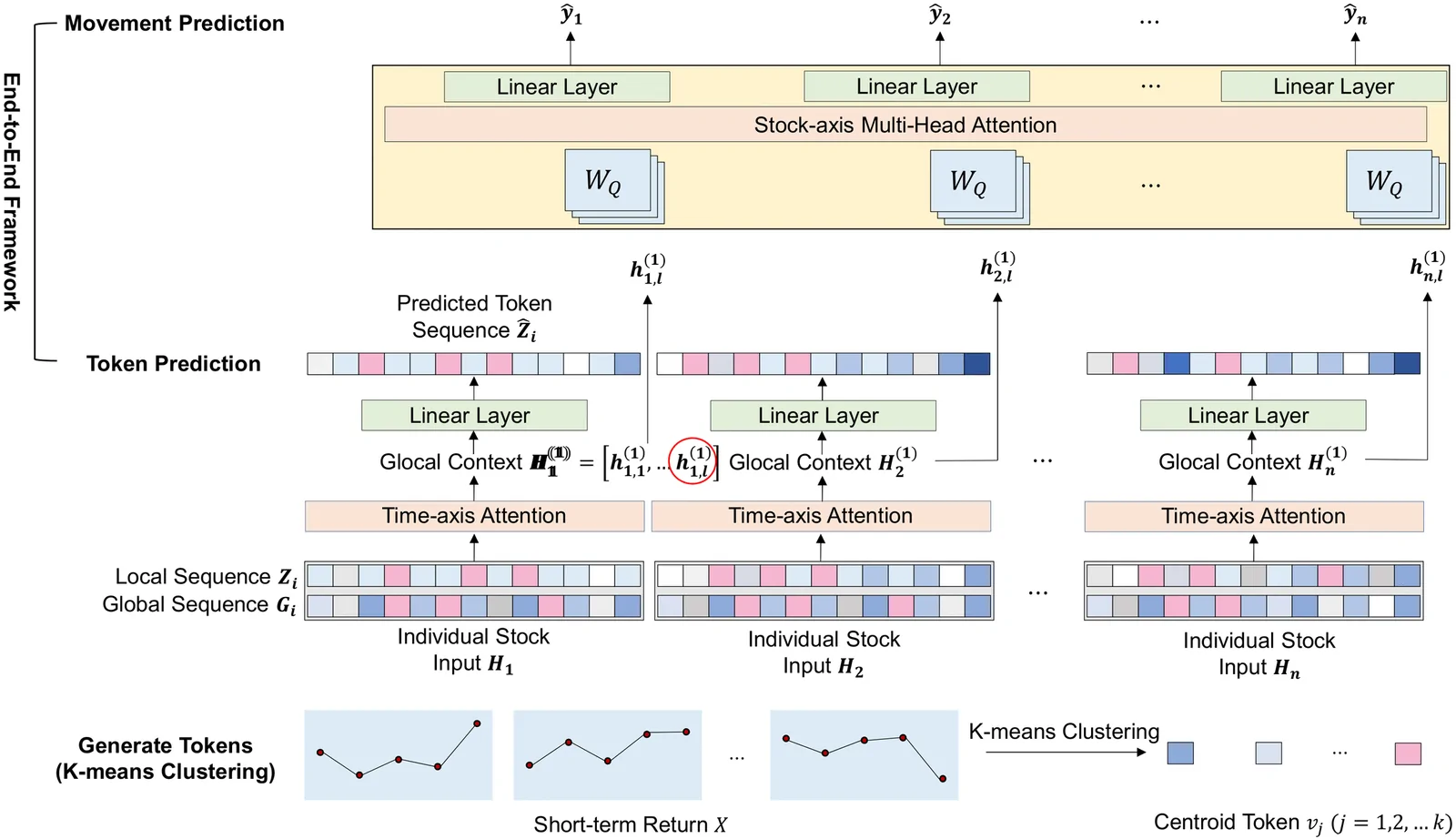
Overview of craft. By converting each stock’s short-term relative returns into discrete tokens, Craft alleviates inherent randomness and highlights short-term patterns. For each time step, a *dynamic global context* sequence is constructed from stock-level correlations and then combined with the local token sequence of each stock, yielding refined next-token features. Finally, the *Stock-axis Multi-Head Attention Layer* applies attention over these features across all stocks, learning which stocks contribute the most to each target prediction. This design enables Craft to capture inter-stock influences more precisely and to accurately predict each stock’s subsequent movement.

Our contributions are summarized as follows:

- **Method.** We propose Craft, an accurate stock prediction framework. Recent successes in large language models (e.g., GPT) show that a decoder operating on coherent, tokenized sequences can capture generalized patterns more effectively. Motivated by this insight, Craft transforms raw returns into stable tokens, allowing the decoder to focus on generalized patterns in stock movements rather than being confounded by high-frequency noise.
- **Accuracy.** We conduct extensive evaluations using major real-world stock market datasets (from the United States, China, Europe, and Japan). Craft achieves the highest accuracy in twelve of the fourteen test settings and ranks the second in the other two, improving accuracy by up to 4.1%p and the Matthews correlation coefficient by up to 7.8 points relative to existing baselines.
- **Case study.** Through visual inspection, we verify that the *temporal stock embeddings* accurately reflect real-world sector relationships, enabling stocks with similar characteristics at each time step to reference one another. This mechanism allows Craft to incorporate a robust *temporal global context*, leveraging time-varying cross-stock correlations for improved modeling.

The symbols used in this paper is in Table 1. The code is available at https://github.com/snudatalab/CRAFT, and the datasets are deposited at https://doi.org/10.5281/zenodo.21506172.

**Table 1:** Symbols used throughout this paper.

Symbol	Description
$p_{i,t}$	Price of stock *i* at time *t*.
$y_{i,t}$	Binary indicator {0,1} for the up or down movement of stock *i* at time *t*.
$\mu ,\epsilon_{t}$	Drift term μ and normally distributed shock $\left(\epsilon_{t}\sim 𝒩(0,\sigma^{2})\right)$ in $p_{t}=p_{t-1}+\mu +\epsilon_{t}$.
$x_{i,t}$	(w−1)-dimensional relative return vector for stock *i* at time *t*, from *w* consecutive prices $\left(p_{t-w+1},...,p_{t}\right)$.
$v_{j}$	*j*-th centroid in ${\mathbb{R}}^{\;w-1}$ (for j=1,...,k), also serving as a token embedding.
$z_{i,t}$	Assigned centroid embedding for stock *i* at time *t*, i.e., $z_{i,t}=v_{\phi (x_{i,t})}$.
$Z_{i,t}$	Sequence $\left[z_{i,(t-\ell +1)},...,z_{i,t}\right]$ of length ℓ, capturing stock *i*’s local token patterns.
$e_{i,t}$	Stock embedding for stock *i* at time *t*, derived from $\Omega_{t}$ via SVD or a similar method.
$g_{i,t}$	Weighted global embedding for stock *i* at time *t*, aggregating embeddings of similar stocks.
$G_{i,t}$	Sequence $\left[g_{i,t-\ell +1},...,g_{i,t}\right]$ of length ℓ.
${\hat{y}}_{i,t}$	Predicted token (label) for stock *i* at time *t* (single-step notation for clarity).

### Related works

We introduce related works on stock movement prediction in two categories based on how they handle inherent randomness of stock data. The first category is *data-centric*, encompassing multi-modal integration and feature engineering. The second category is *model-centric*, which emphasizes inference architectures tailored for financial time-series forecasting or exploiting financial market characteristics [10,11].

### Data augmentation

Data augmentation has been widely used to improve predictive accuracy with additional information. In stock forecasting, such augmentation is motivated by the challenge of predicting price movements from limited or partially informative historical data.

One widely adopted technique is to enhance input features of price series. Zhang [12] and Khandelwal et al. [13] combine ARIMA [14] with neural networks, but purely autocorrelation-based methods cannot fully capture complex market dynamics. Another frequent approach is engineering technical indicators [2] or factor modeling using fundamental ratios [1,15], which can be effective but often rely on linear assumptions that fail under nonstationary conditions [16].

More recently, researchers have actively integrated alternative modalities to capture broader market contexts. For instance, pre-trained language models (LLMs) are increasingly utilized to extract rich representations for general time-series forecasting [17,18]. Concurrently, graph-based augmentations employ concept-oriented networks to explicitly model inter-stock correlations [19], and hypergraph neural networks extend this idea to capture higher-order relationships among stocks [20].

Although these data-centric strategies can sometimes improve short-term forecasts, they inevitably retain much of the randomness intrinsic to stock prices. Moreover, if the augmented feature space grows faster than the available data, the curse of dimensionality may obscure patterns in the data. This persistent challenge motivates our approach, which relies exclusively on historical price data to reduce randomness directly by focusing on short-term patterns, thereby avoiding complexities on external variables.

### Model refinement

A complementary line of research aims to address the inherent randomness in stock data by refining the predictive model architecture. Since stock prices form a time series, many studies [21,22] leverage RNNs to capture sequential dependencies. Early GRU/LSTM networks helped mitigate vanishing and exploding gradients, enabling more stable training. However, they still exhibit critical limitations:

- **Propagation of randomness.** When data contain substantial noise and nonstationary characteristics, more recurrent layers may propagate, rather than reduce, randomness.
- **Long-range dependencies.** Even with gating mechanisms, it is difficult to capture multiple time scales in financial data.

In recent years, Transformer-based models have garnered attention for stock price forecasting. Although conceptually similar to attention-augmented RNNs, Transformers remove recurrence entirely and rely on self-attention to capture local and global dependencies in parallel. Their positional encoding compensates for the lack of sequence ordering in self-attention [23].

Several works exploit Transformer structures: DTML (Data-axis Transformer with Multi-Level contexts) [11] utilizes attentive contexts across time and stock axes, TEGRU (Transformer Encoder Gated Recurrent Unit) [24] integrates news sentiments, and MASTER (MArket-guided Stock TransformER) [9] models cross-time correlations with feature selection. Beyond movement prediction, Transformer variants such as Informer and Autoformer have been benchmarked for pairs trading on equity and cryptocurrency markets [25]. While these methods often improve robustness, large model sizes and deep layers risk amplifying random fluctuations, making it hard to overcome the volatility of financial markets [26].

In contrast, our proposed Craft reduces noise earlier in the process using centroid-based tokenization and employs an *end-to-end* architecture that integrates self-attention across both time axis and stock axis. This approach minimizes the impact of random fluctuations while maintaining predictive accuracy. Rather than stacking numerous layers on raw price data, Craft focuses on short-term local patterns and inter-stock correlations, mitigating hidden-representation noise and capturing crucial temporal signals in dynamic markets.

### Proposed method

We propose Craft (Centroid-based Randomness Smoothing Approach for Stock Forecasting with Transformer Architecture), an accurate stock movement prediction method. Our formal problem definition is as follows.

**Problem 1** (Stock movement prediction.)***. Given*** *the historical price vector* $[\;p_{i,t-w+1},\;p_{i,t-w+2},\;...,\;p_{i,t}{]}^{⊤}\;\in \;{\mathbb{R}}^{w}$ *for an individual stock i across multiple stocks over a lookback window w ending at the current time step t,* ***predict*** *the discrete price movement* $y_{i,t+1}\in \{0,1\}$ *at day t + 1, where* $y_{i,t+1}=1$ *if* $p_{i,t+1}>p_{i,t}$ *and 0 otherwise.*

### Overview

#### Challenges and ideas.

Raw price data often exhibit notable volatility, as different market participants assign varying valuations to the same asset. Inspired by large language models (LLMs) [27,28] which tokenizes text into coherent units for next-token prediction, Craft formulates this forecasting process by transforming continuous, noisy price segments into stable, discrete representations to uncover consistent market dynamics. To effectively solve this task, we identify three main challenges and propose the corresponding solutions:

**C1 How can we extract clear patterns from data with inherent randomness?**
  Stock prices often fluctuate; this randomness can mask meaningful local patterns crucial for accurate predictions.
**C2 How can we consider complex and dynamic inter-stock correlations to enhance predictive power?**
  Even after identifying local patterns for each individual stock, overall movements can still be influenced by wider market or sector-based trends. A framework is needed to integrate correlated stock signals effectively.
**C3 How can we utilize dynamic correlation patterns for accurate price movement prediction?**
  Historical correlation matrices capture average relationships, but actual cross-stock dependencies shift over time. Leveraging evolving correlations alongside local price patterns remains a key challenge for accurate price movement prediction.
  We address the above challenges with the following three ideas.

#### I1 Centroid-based price embedding (Token generation).

To address **C1**, Craft clusters noisy return data into short-term “centroid-based” tokens, analogous to how large language models tokenize text. By mitigating high-frequency volatility, we obtain coherent local price segments that clarify time dependencies.

#### I2 Related context aggregation via dynamic stock embedding (Related context aggregation via dynamic stock embedding).

To address **C2**, Craft computes correlation-based stock embeddings at each time step and forms a global context that aggregates signals from correlated stocks. This allows Craft to capture inter-stock dependencies and unify local patterns with broader market influences.

#### I3 Two-stage attention in an end-to-end framework (Inter-stock glocal context aggregation via end-to-end framework).

To address **C3**, Craft employs a dual attention mechanism. A temporal-axis attention captures local time dependencies and predicts the next token for each stock. A subsequent stock-axis attention integrates cross-stock correlations for refined price movement predictions. By training these attention modules in a joint manner, the model balances temporal and cross-stock patterns.

The overall procedure of Craft (Fig 1) consists of three main modules. First, the Token Generation module takes the raw price vectors as input, applies relative normalization, and uses *k*-means clustering to convert them into a stable sequence of centroid tokens (**I1**). Subsequently, the model extracts temporal stock embeddings via SVD to construct a dynamic global context for each stock, which is then processed alongside the local tokens through a time-axis self-attention mechanism to capture temporal dependencies (**I2**). Finally, the representations of all stocks at the last time step are fed into a stock-axis multi-head self-attention module to exchange information among correlated peers, yielding the final price movement prediction in an end-to-end manner (**I3**).

### Token generation

We transform each short-term price segment into a discrete token. Our objective is to suppress high-frequency randomness and expose stable local patterns to the Transformer. Instead of directly using the noisy raw price returns, our idea is to normalize the short-term price segments across stocks and times, and replace them with representative short-term price patterns where inherent randomness is effectively smoothed.

#### Relative normalization.

Let $p_{i,t}$ be the price of stock *i* at day *t*. We choose a small window size *w* (e.g., fewer than 20 days), and for each 1≤j<w define the *j*-day relative re*t*urn:


$$\begin{array}{c} x_{i,t}^{\left[j\right]}\;=\;\frac{p_{i,\;(t-w+1+j)}}{p_{i,\;(t-w+1)}}, \end{array}$$

capturing the ratio between the price at day (t−w+1+j) and the price at day (t−w+1). This yields a (w−1)-dimensional vector of relative returns:


$$\begin{array}{c} x_{i,t}\;=\;[\;x_{i,t}^{\left[1\right]},\;x_{i,t}^{\left[2\right]},\;...,\;x_{i,t}^{\left[w-1\right]}{]}^{⊤}, \end{array}$$

which offsets scale discrepancies and reduces nonstationary effects.

#### Centroid tokenization.

Stock prices often exhibit significant randomness on top of any baseline trend. A standard idealization is the *random walk with drift*, where an individual asset price evolves as


$$\begin{array}{c} p_{i,t}\;=\;p_{i,t-1}+\mu +\epsilon_{t},\;\epsilon_{t}\sim 𝒩(0,\sigma^{2}). \end{array}$$

To suppress the noise while retaining a small set of recurring short-term patterns, we adopt a vector-quantization scheme analogous to VQ-VAE [29]: each short-term return vector is replaced by its nearest centroid, yielding a discrete token. We apply *k*-means clustering to the set 𝒳 of vectors $x_{i,t}\in {\mathbb{R}}^{\;w-1}$ across all stocks *i* and times *t* within the training period. The resulting cluster centroids are


$$\begin{array}{c} {𝐯}_{1},\;{𝐯}_{2},\;...,\;{𝐯}_{k},\;({𝐯}_{j}\in {\mathbb{R}}^{\;w-1}), \end{array}$$

which we also treat as *token embeddings*. Let


$$\begin{array}{c} \phi (x_{i,t})\;\in \;\{1,...,k\} \end{array}$$

assign each vector $x_{i,t}$ to its nearest centroid. Hence, for each $x_{i,t}$, the final token embedding is


$$\begin{array}{c} z_{i,t}\;=\;{𝐯}_{\phi (x_{i,t})}\;\in \;{\mathbb{R}}^{\;w-1}. \end{array}$$

We use a grid search over k∈{50,100,150,200,250,300,350} to find the optimal *k* separately for each market regime, fitting the centroids on the regime’s training split and selecting *k* on its validation split. The standard elbow [30] and silhouette [31] methods often fail to find stable solutions for noisy stock price data with a large *k* [32,33]. Consequently, we select the *k* that yields the highest average validation accuracy for stock movement prediction. The detailed sensitivity analysis and selection process for *k* are presented in the Experiment section.

#### Centroid-token sequence.

After assigning $x_{i,t}$ to a token embedding $z_{i,t}$ for each stock *i* and day *t*, we form a *sequence* of centroid tokens:


$$\begin{array}{c} Z_{i,t}\;=\;[\;z_{i,\;t-\ell +1},...,z_{i,t}]\;\in \;{\mathbb{R}}^{\ell \times (w-1)}. \end{array}$$

Here, ℓ is the *sequence length* that the Transformer decoder uses as the number of tokens.

#### Next-token prediction reframing.

Instead of forecasting the raw price $p_{i,t+1}$, we aim to predict $z_{i,t+1}\;\in \;\{{𝐯}_{1},...,{𝐯}_{k}\},$ which parallels “next-token” modeling in language tasks. This lets the Transformer attend to stable local patterns rather than random spikes in the original time series.

In practice, a relatively small window size *w* (fewer than 20 trading days) further reduces high-frequency fluctuations, so these locally consistent tokens provide robust features for downstream prediction and portfolio selection.

### Related context aggregation via dynamic stock embedding

We construct a time-varying global context for each stock from temporal cross-stock correlations. Inter-stock dependencies change with market regimes and sector rotations. Per-stock signals or static factor loadings do not represent the contemporaneous market structure. We obtain spectral embeddings via Eigendecomposition of a rolling cross-stock correlation matrix and use them to aggregate related stocks at each time step. This follows the factor-model tradition of extracting latent factors from return correlations [1,15].

#### Daily return.

For each stock *i* at day *t*, let


$$\begin{array}{c} r_{i,t+1}\;=\;\frac{p_{i,t+1}}{p_{i,t}} \end{array}$$

be the daily return of *i* at *t* which captures the change of the daily closing price.

#### Temporal stock embedding via SVD.

Let *N* be the number of stocks in our universe. At each time *t*, we collect the daily returns of the 60 trading days up to t−1 (roughly 3 months) into a matrix


$$\begin{array}{c} {𝐑}_{t}\in {\mathbb{R}}^{N\times 60}, \end{array}$$

where the *i*-th row, ${𝐫}_{i,t}^{⊤}$, corresponds to stock *i*’s returns over that period. We then construct an N×N correlation matrix:


$$\begin{array}{c} \Omega_{t}\;=\;Corr({𝐑}_{t}). \end{array}$$ (1)

where each entry $\Omega_{t}[i,j]$ is the sample correlation coefficient between the past 60 daily returns of stocks *i* and *j* (days t−60 to t−1) at time *t*. Applying SVD to $\Omega_{t}$ yields


$$\begin{array}{c} \Omega_{t}\;=\;{𝐔}_{t}\;\Sigma_{t}\;{𝐔}_{t}^{⊤}, \end{array}$$ (2)

where ${𝐔}_{t}\in {\mathbb{R}}^{N\times d_{e}}$ contains the top $d_{e}$ left singular vectors. We define the *stock embedding matrix* ${𝐄}_{t}={𝐔}_{t}$, so the embedding for stock *i* at time *t* is the *i*-th row of ${𝐄}_{t}$, denoted as ${𝐞}_{i,t}^{⊤}$.

#### Global context via embedding similarities.

To incorporate inter-stock relationships, we derive a global context ${𝐠}_{i,t}\in {\mathbb{R}}^{\;w-1}$ for each stock *i* by aggregating embeddings of other stocks j≠i. Specifically, let


$$\begin{array}{c} {𝐠}_{i,t}\;=\;\sum_{j\neq i}\alpha_{ij,t}\;{𝐳}_{j,t},\;\alpha_{ij,t}\;=\;\frac{\exp\;(-\;\|{𝐞}_{i,t}-{𝐞}_{j,t}{\|}^{2})}{\sum_{k\neq i}\exp\;(-\;\|{𝐞}_{i,t}-{𝐞}_{k,t}{\|}^{2})}. \end{array}$$ (3)

Here, $\left\|{𝐞}_{i,t}-{𝐞}_{j,t}\right\|$ is the Euclidean distance between embeddings for stocks *i* and *j*, and $\alpha_{ij,t}$ normalizes these distances so that $\sum_{j}\alpha_{ij,t}=1$. This ensures that related (similar) stocks contribute more strongly to the context of stock *i*. Both ${𝐞}_{i,t}$ and ${𝐞}_{j,t}$ are rows of the same matrix ${𝐔}_{t}$, so any column-wise sign flip from the SVD affects both rows equally and cancels inside the difference ${𝐞}_{i,t}-{𝐞}_{j,t}$.

#### Time-axis self-attention.

We predict future price patterns for each stock *i* from:

1. local token sequence ${𝐙}_{i,t}\in {\mathbb{R}}^{\ell \times (w-1)}$ (from Token generation), and
2. global context sequence ${𝐆}_{i,t}=[{𝐠}_{i,t-\ell +1},...,{𝐠}_{i,t}]\in {\mathbb{R}}^{\ell \times (w-1)}.$

We concatenate these along the feature dimension:


$$\begin{array}{c} {𝐇}_{raw}\;\in \;{\mathbb{R}}^{\;\ell \times \;2(w-1)\;}. \end{array}$$

Next, we apply a linear projection via a trainable matrix ${𝐖}_{proj}\in {\mathbb{R}}^{\;2(w-1)\times d}$ to refine feature representations and unify the dimension:


$$\begin{array}{c} 𝐇\;=\;{𝐇}_{raw}\;{𝐖}_{proj}\;\in \;{\mathbb{R}}^{\;\ell \times d}. \end{array}$$

Craft adopts a *decoder-only* masked self-attention approach, ensuring that for the τ-th token in a sequence, subsequent tokens (τ+1,...) remain hidden.

#### Self-attention operation.

We project 𝐇 into queries, keys, and values:


$$\begin{array}{c} 𝐐=𝐇{𝐖}_{Q},\;𝐊=𝐇{𝐖}_{K},\;𝐕=𝐇{𝐖}_{V}, \end{array}$$

where ${𝐖}_{Q},{𝐖}_{K},{𝐖}_{V}\in {\mathbb{R}}^{d\times d}$. The attention output is


$$\begin{array}{c} {\tilde{𝐇}}^{\left(1\right)}\;=\;softmax\;(\frac{𝐐\;{𝐊}^{⊤}}{\sqrt{d}})\;𝐕. \end{array}$$ (4)

Next, we apply a residual connection and an MLP to obtain


$$\begin{array}{c} {𝐇}^{\left(1\right)}\;=\;MLP\;(𝐇+{\tilde{𝐇}}^{\left(1\right)})\;\in \;{\mathbb{R}}^{\ell \times d}. \end{array}$$ (5)

Here, *d* is the dimension used to represent temporal contexts. This representation encodes temporal dependencies at each time step. Moreover, ${𝐇}^{\left(1\right)}$ reflects both individual stock’s local patterns and related stocks’ global context, thus providing a “glocal” representation essential for accurate forecasting.

#### Token prediction.

Let $c_{\tau }\in \{1,...,k\}$ be the correct token label at time step τ. We use a linear projection to produce ${\hat{c}}_{\tau }\in \{1,...,k\}$ with probability $\hat{P}({\hat{c}}_{\tau }=j)$ for each time step τ. Let ${𝐡}_{\tau }^{\left(1\right)}\in {\mathbb{R}}^{d}$ be the τ-th row of ${𝐇}^{\left(1\right)}$. Then, the probability of the next token being *j* is given by


$$\begin{array}{c} \hat{P}({\hat{c}}_{\tau }=j)=softmax({𝐖}_{\text{out}}\;{𝐡}_{\tau }^{\left(1\right)}{)}_{j},\;{𝐖}_{\text{out}}\in {\mathbb{R}}^{k\times d}. \end{array}$$

#### Next token prediction loss.

We define the time-axis classification loss as the cross-entropy over centroid labels:


$$\begin{array}{c} L_{\text{time}}=CE({\hat{c}}_{\tau },\;c_{\tau }), \end{array}$$ (6)

and sum over all time steps. Here, no additional token-embedding training is required, because $\left\{{𝐯}_{1},...,{𝐯}_{k}\right\}$ have been trained with *k*-means as centroids.

### Inter-stock glocal context aggregation via end-to-end framework

We aggregate cross-stock information along the stock axis to produce final price movement predictions from a glocal (global + local) representation. Methods that rely only on per-stock information or assume fixed correlations miss time-varying relations [8,10,34]. Existing cross-stock attention models [9,11] typically focus on exchanging intermediate hidden contexts. However, Craft takes a distinct approach by leveraging a glocal context to directly model realistic market correlations among the final price movement representations. Specifically, we stack the last step vectors of all stocks and apply multi-head self-attention along the stock axis to exchange information among correlated peers. We train this classifier jointly with the time-axis next-token objective in an end-to-end framework.

#### Next price movement prediction.

Recall that each stock *i* has a time-axis representation


$$\begin{array}{c} {𝐇}_{i}^{\left(1\right)}=[\begin{array}{c} {𝐡}_{i,1}^{(1)⊤}\\ {𝐡}_{i,2}^{(1)⊤}\\ \vdots \\ {𝐡}_{i,\ell }^{(1)⊤} \end{array}]\in {\mathbb{R}}^{\ell \times d}. \end{array}$$

obtained from Related context aggregation via dynamic stock embedding. We focus on the final time step’s output ${𝐡}_{i,\ell }^{\left(1\right)}\in {\mathbb{R}}^{d}$, which captures the most recent local context for stock *i*. Collecting these vectors from all *N* stocks yields


$$\begin{array}{c} {𝐇}_{N}^{\left(1\right)}=[\begin{array}{c} {𝐡}_{1,\ell }^{(1)⊤}\\ {𝐡}_{2,\ell }^{(1)⊤}\\ \vdots \\ {𝐡}_{N,\ell }^{(1)⊤} \end{array}]\in {\mathbb{R}}^{N\times d}. \end{array}$$

This matrix serves as the input to a *stock-axis* multi-head self-attention, allowing Craft to refine each stock’s direction forecast by leveraging the correlations among these final-step embeddings.

#### Stock-axis self-attention.

Let *M* be the number of heads, and let $d_{1}=\frac{d}{M}$ be the dimension per head. We project ${𝐇}_{N}^{\left(1\right)}$ into queries, keys, and values for each head m∈{1,...,M}:


$$\begin{array}{c} {𝐐}_{m}={𝐇}_{N}^{\left(1\right)}\;{𝐖}_{Q}^{\left(m\right)},\;{𝐊}_{m}={𝐇}_{N}^{\left(1\right)}\;{𝐖}_{K}^{\left(m\right)},\;{𝐕}_{m}={𝐇}_{N}^{\left(1\right)}\;{𝐖}_{V}^{\left(m\right)}, \end{array}$$

where ${𝐖}_{Q}^{\left(m\right)},{𝐖}_{K}^{\left(m\right)},{𝐖}_{V}^{\left(m\right)}\in {\mathbb{R}}^{d\times d_{1}}$. The scaled dot-product attention is


$$\begin{array}{c} {\tilde{𝐇}}_{m}^{\left(2\right)}=softmax\;(\frac{{𝐐}_{m}\;{𝐊}_{m}^{⊤}}{\sqrt{d_{1}}})\;{𝐕}_{m}. \end{array}$$ (7)

We concatenate these *M* outputs along the feature dimension:


$$\begin{array}{c} {\tilde{𝐇}}_{\text{cat}}^{\left(2\right)}=[{\tilde{𝐇}}_{1}^{\left(2\right)}\;\|\;\cdots \|\;{\tilde{𝐇}}_{M}^{\left(2\right)}]\in {\mathbb{R}}^{N\times (M\;d_{1})}={\mathbb{R}}^{N\times d}, \end{array}$$

We apply a linear transformation ${𝐖}_{O}\in {\mathbb{R}}^{d\times d}$ for refinement and add a residual connection:


$$\begin{array}{c} {\tilde{𝐇}}^{\left(2\right)}={\tilde{𝐇}}_{\text{cat}}^{\left(2\right)}\;{𝐖}_{O},\;{𝐇}^{\left(2\right)}={𝐇}_{N}^{\left(1\right)}+{\tilde{𝐇}}^{\left(2\right)}. \end{array}$$ (8)

Let ${𝐡}_{i}^{\left(2\right)}\in {\mathbb{R}}^{d}$ be the *i*-th row of ${𝐇}^{\left(2\right)}$. Finally, we map each ${𝐡}_{i}^{\left(2\right)}$ to a 2-dimensional logit vector


$$\begin{array}{c} {𝐨}_{i}={𝐖}_{F}\;{𝐡}_{i}^{\left(2\right)},\;{𝐖}_{F}\in {\mathbb{R}}^{2\times d}, \end{array}$$

then apply a softmax:


$$\begin{array}{c} softmax({𝐨}_{i})=[p({\hat{y}}_{i,t+1}=1),\;p({\hat{y}}_{i,t+1}=0)]. \end{array}$$

Here, $p({\hat{y}}_{i,t+1}=1)$ is the probability that stock *i*’s next return is predicted to be positive.

#### Next price movement prediction loss.

Let $y_{i,t+1}\in \{0,1\}$ be the ground-truth upward/downward label of stock *i* at time *t* + 1, and ${\hat{y}}_{i,t+1}\in \{0,1\}$ be the predicted class. We define


$$\begin{array}{c} L_{\text{stock}}=-\sum_{i=1}^{N}[y_{i,t+1}\;\log\;p({\hat{y}}_{i,t+1}=1)+(1-y_{i,t+1})\;\log\;p({\hat{y}}_{i,t+1}=0)]. \end{array}$$

#### Total prediction loss.

Combining the time-axis loss *L*_time_ (from Related context aggregation via dynamic stock embedding) with the stock-axis loss gives


$$\begin{array}{c} L_{\text{total}}=L_{\text{time}}+\lambda_{\text{stock}}\;L_{\text{stock}}. \end{array}$$

where $\lambda_{\text{stock}}$ balances next-token prediction and final price movement classification. By minimizing *L*_total_, our end-to-end framework learns ${𝐇}^{\left(1\right)}$ for accurate centroid-token forecasting, while ${𝐇}^{\left(2\right)}$ leverages inter-stock attention for robust price movement prediction.

## Experiments

We conduct extensive experiments on real-world datasets to provide answers to the following questions.

**Q1. Stock price movement prediction performance (see Stock price movement prediction performance (Q1)).** How accurate is Craft compared to previous state-of-the-art competitors?
**Q2. Investment simulation (see Investment simulation (Q2)).** Can Craft generate profitable returns, and does it maintain consistent performance across stock markets in multiple countries?
**Q3. Stock embedding quality (see Stock embedding quality (Q3)).** Does the stock embedding, derived via SVD for Craft’s dynamic global context, effectively capture correlated stocks so that their price information is reflected accordingly?
**Q4. Ablation study (see Ablation study (Q4)).** How do the key modules of Craft contribute to improvements in predictive performance?

## Experimental settings

### Datasets

We use six datasets evaluated across three distinct market regimes, as summarized in Table 2: the COVID-19 Crisis, the AI Rally, and the Recent Volatile period. Notably, the experiments for the Recent Volatile period comprehensively include both the newly proposed datasets and the existing public benchmarks. For the public datasets, the AAAI24 dataset is employed by Li et al. [9] in a data augmentation approach that leverages external macro data, while the CIKM24 dataset is used by Cao et al. [8] for an incremental learning-based model refinement methodology. We adopt the preprocessed versions directly from their official repositories, retaining the same training, validation, and test splits. SP500, CSI300, NI225, and EURO50 are new public benchmark datasets that we collect from the Yahoo Finance public feed via the yfinance Python library, covering the US, China, Japan, and Europe stock markets, respectively. Downloads were finalized in March 2025. We use the split- and dividend-adjusted closing prices. To avoid potential sector biases or small-cap effects, we select the union of the top 20% of stocks by market capitalization or by trading volume within each sector, restricted to stocks continuously listed from 2020−01 through the download date so that the dataset contains no delisting event, forming our final dataset. This procedure yields a more representative and stable set of stocks for our datasets. The three regime-specific test windows are nested temporal splits of this single fixed universe. This continuous-listing filter introduces survivorship bias, but applies uniformly to all evaluated methods, so the relative performance ranking among models is not affected by this universe choice.

**Table 2:** Summary of datasets and temporal splits across three distinct market regimes.

Dataset (Region)	Train	Validation	Test
**1. COVID-19 Crisis Period**			
**Four New Datasets** ^3^	2013/01 ~	2019/10–2019/12	2020/01–2020/03
**2. AI Rally Period**			
**Four New Datasets** ^3^	2020/04 ~	2023/10–2023/12	2024/01–2024/06
**3. Recent Volatile Period (Overall)**			
*(Public Datasets)*			
**CIKM24 (US)** ^1^	2008/01 ~	2020/04–2020/06	~ 2023/12
**AAAI24 (China)** ^2^	2008/01 ~	2015/01–2016/12	~ 2020/12
*(New Datasets)* ^3^			
**SP500 (US)**	2021/01 ~	2022/10–2023/11	~ 2024/12
**CSI300 (China)**	2020/04 ~	2024/01–2024/05	~ 2024/12
**NI225 (Japan)**	2020/01 ~	2023/07–2024/07	~ 2024/12
**EURO50 (Europe)**	2020/04 ~	2024/01–2024/04	~ 2024/12

^1^https://github.com/caozhiy/MATCC ^2^https://github.com/SJTU-DMTai/MASTER ^3^https://doi.org/10.5281/zenodo.21506172

### Competitors

We compare Craft with the following baselines for stock movement prediction:

- **LSTM** [34] is a simple baseline that employs a standard LSTM to predict stock movements.
- **ALSTM** [35] incorporates temporal attentive contexts into an LSTM, highlighting the most influential time steps for price predictions.
- **PatchTST** [36] is a general time-series forecasting method that converts the input into short-term patches and leverages a Transformer architecture for effective prediction.
- **Informer** [37] is a long-sequence forecasting Transformer that uses ProbSparse self-attention to reduce the quadratic attention cost.
- **Autoformer** [38] replaces dot-product attention with an auto-correlation mechanism and an internal series-decomposition block.
- **FEDformer** [39] performs attention in the frequency domain and adds a mixture-of-experts decomposition of trend and seasonal components.
- **MATCC** [8] explicitly extracts overarching market trends and decomposes stock data into trend and fluctuation components, further exploiting cross-time correlations for robust prediction.
- **MASTER** [9] is a market-guided stock Transformer that captures momentary, cross-time stock correlations and leverages market information for automatic feature selection.
- **MA+LSTM**, **EMA+LSTM**, **ARIMA+LSTM**, **Wavelet+LSTM**, and **Kalman+LSTM** apply a denoising or smoothing step (moving average, exponential moving average, ARIMA, wavelet thresholding, or a Kalman filter) to the raw prices before a standard LSTM.
- **ARMA**, **ARIMA-GARCH** (the sign of the one-step forecast), **Momentum**, and **Reversal** are classical statistical and factor baselines.

Informer, Autoformer, and FEDformer are regression forecasters. To fit the movement-prediction setting, we append a sigmoid output layer that converts their forecasts into up/down probabilities.

The five preprocessing+LSTM baselines isolate the effect of denoising alone, which Craft’s centroid tokenization is designed to surpass. Each smoothing step uses a standard daily-frequency configuration fixed across all datasets: a 5-day simple/exponential moving-average window [40], an ARIMA(1,1,1) specification [14,41], Haar wavelet denoising at two levels with soft universal thresholding [42,43], and a local-level Kalman filter with maximum-likelihood noise variances [44]. Fixing these common defaults rather than tuning per dataset isolates the smoothing mechanism and avoids a per-dataset tuning advantage for the denoising baselines. The four classical baselines (ARMA, ARIMA-GARCH, momentum, and reversal) are deterministic, so each is reported from a single run without a standard deviation.

### Evaluation metrics

We report the mean and standard deviation over five random seeds (from 0 to 4) for ACC and MCC. We use four evaluation metrics:

- **Accuracy (ACC)** is the fraction of daily movements correctly predicted by each model.
- **Matthews Correlation Coefficient (MCC)** is a balanced metric that takes into account the overall distribution of correct predictions. Given the counts of true positives, true negatives, false positives, and false negatives, MCC is defined as $\frac{tp\times tn\;-\;fp\times fn}{\sqrt{\left(tp+fp)(tp+fn)(tn+fp)(tn+fn\right)}}$ where *tp* and *tn* refer to correctly predicted upward and downward movements, and *fp* and *fn* denote incorrect predictions.
- **Annualized Sharpe Ratio (ASR)** is the annualized ratio of the sample mean of daily test returns to the sample standard deviation of those returns over the test period.
- **Relative Maximum Drawdown (RMDD) is the largest percentage drop from a running peak of the cumulative return over the test period.**

#### Hyperparameters.

We search the hyperparameters of Craft as follows: the input data length w∈{5,10,20}, the input token sequence length ℓ∈{5,10,20}, the hidden layer size d∈{64,128,256}, the number of attention layers ∈{1,2,4}, the number of attention heads ∈{1,2,4}, the loss balancing parameter λ∈{0.1,0.3,0.5,0.7,0.9}, and the learning rate ∈{0.01,0.001,0.0001}. The SVD stock-embedding dimension is fixed at $d_{e}=10$ for all datasets rather than searched. Among these, the hidden layer size *d*, the loss balancing λ, and the learning rate η exert the strongest influence on Craft’s validation performance. S1 Table reports the measured final-epoch magnitudes of the two loss terms, which sit on clearly different scales and are balanced by λ. We report their ACC and MCC sensitivity profiles in Figs 2–7. Guided by these profiles, we select per regime a parameter set that performs robustly across the evaluated datasets, favoring general-purpose choices over extreme grid points. For competing methods, we simply use their default settings from the publicly available repositories. MASTER and MATCC further receive their original input features, namely the Alpha158 stock indicators together with the 63-dimensional market-feature block that their architectures consume, computed from each dataset’s own market index. Craft instead uses returns alone. Complete training details and per-dataset selected hyperparameters are documented in the released code at https://github.com/snudatalab/CRAFT.

**Fig 2:**
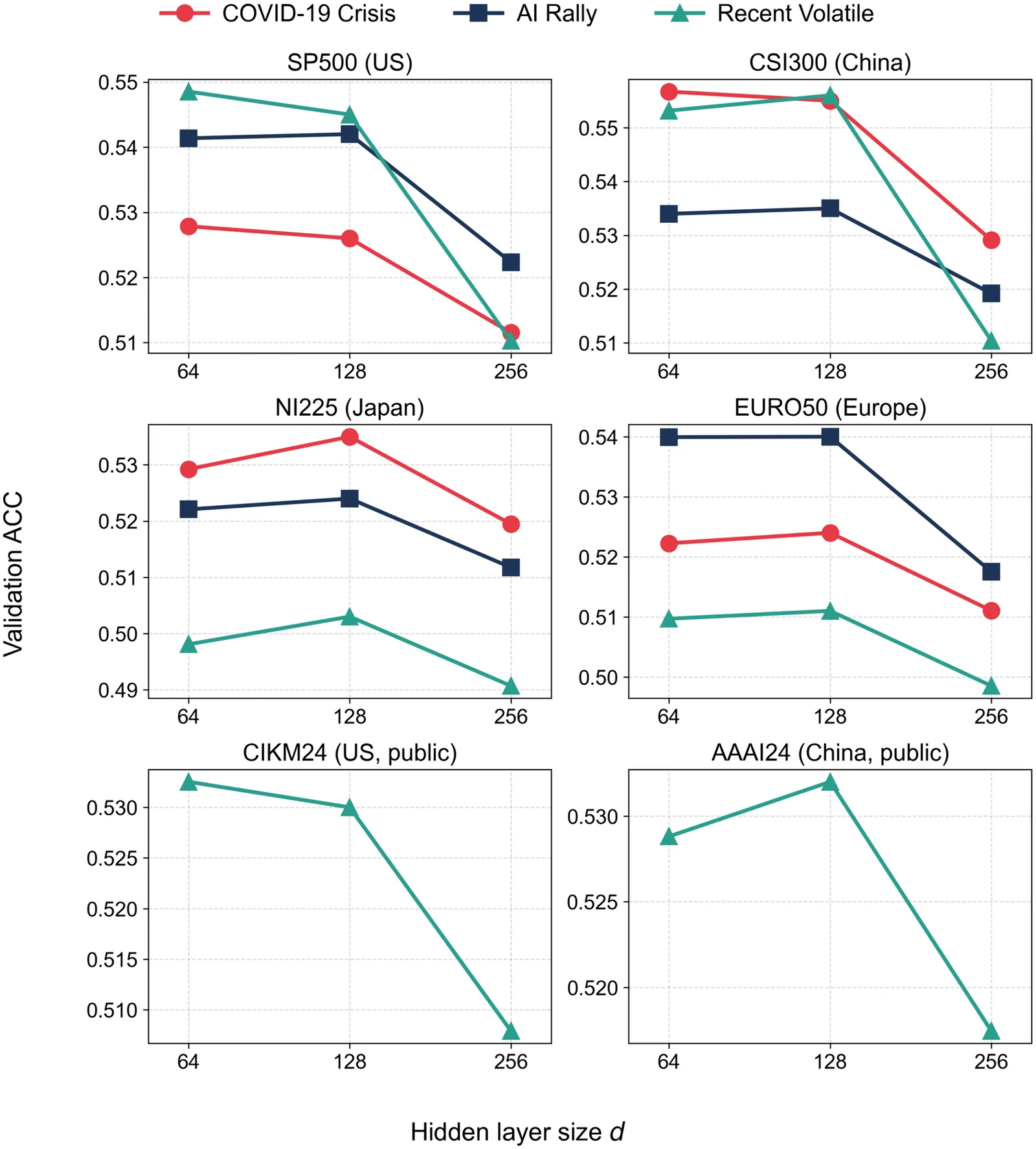
Validation-ACC sensitivity of craft to the hidden layer size *d.* Each panel corresponds to one dataset. Lines distinguish the three evaluated test regimes for our four new datasets (top two rows). CIKM24 and AAAI24 (bottom row) are evaluated only under the Recent Volatile regime because their fixed test windows do not cover the COVID-19 Crisis or AI Rally periods (cf. Table 2). The remaining hyperparameter-sensitivity figures use the same panel layout.

**Fig 3:**
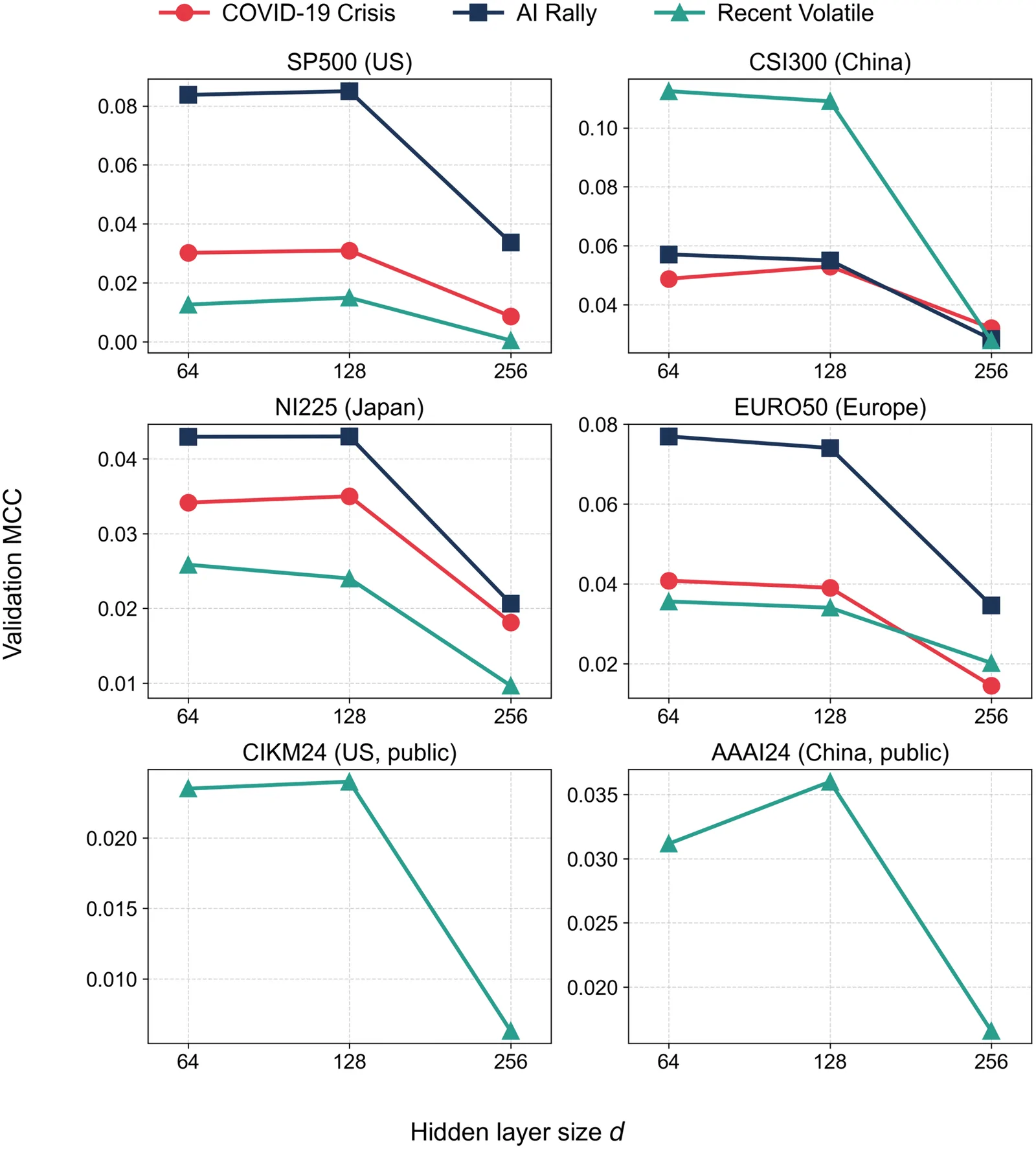
Validation-MCC sensitivity of craft to the hidden layer size *d.*

**Fig 4:**
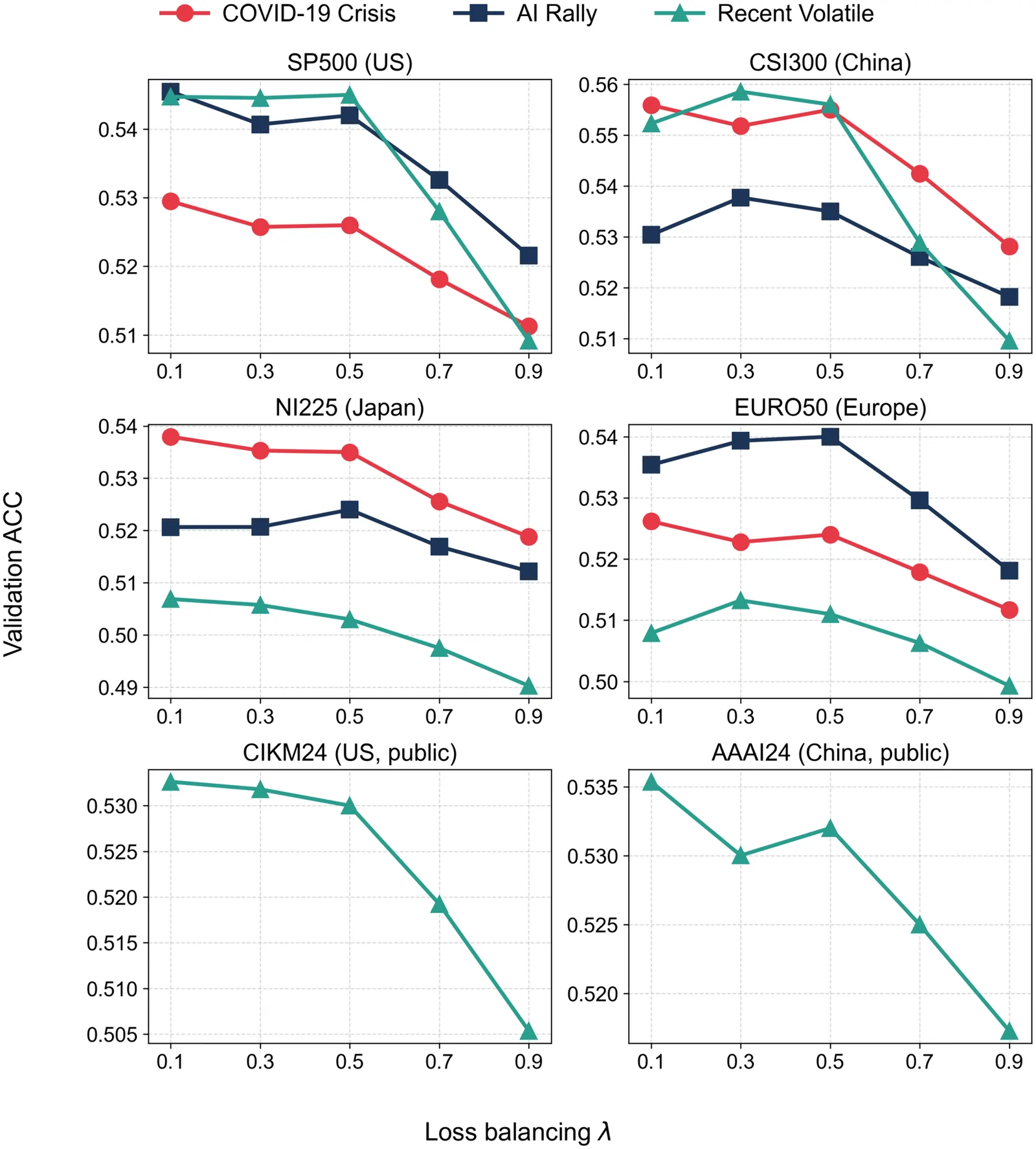
Validation-ACC sensitivity of craft to the loss balancing parameter λ.

**Fig 5:**
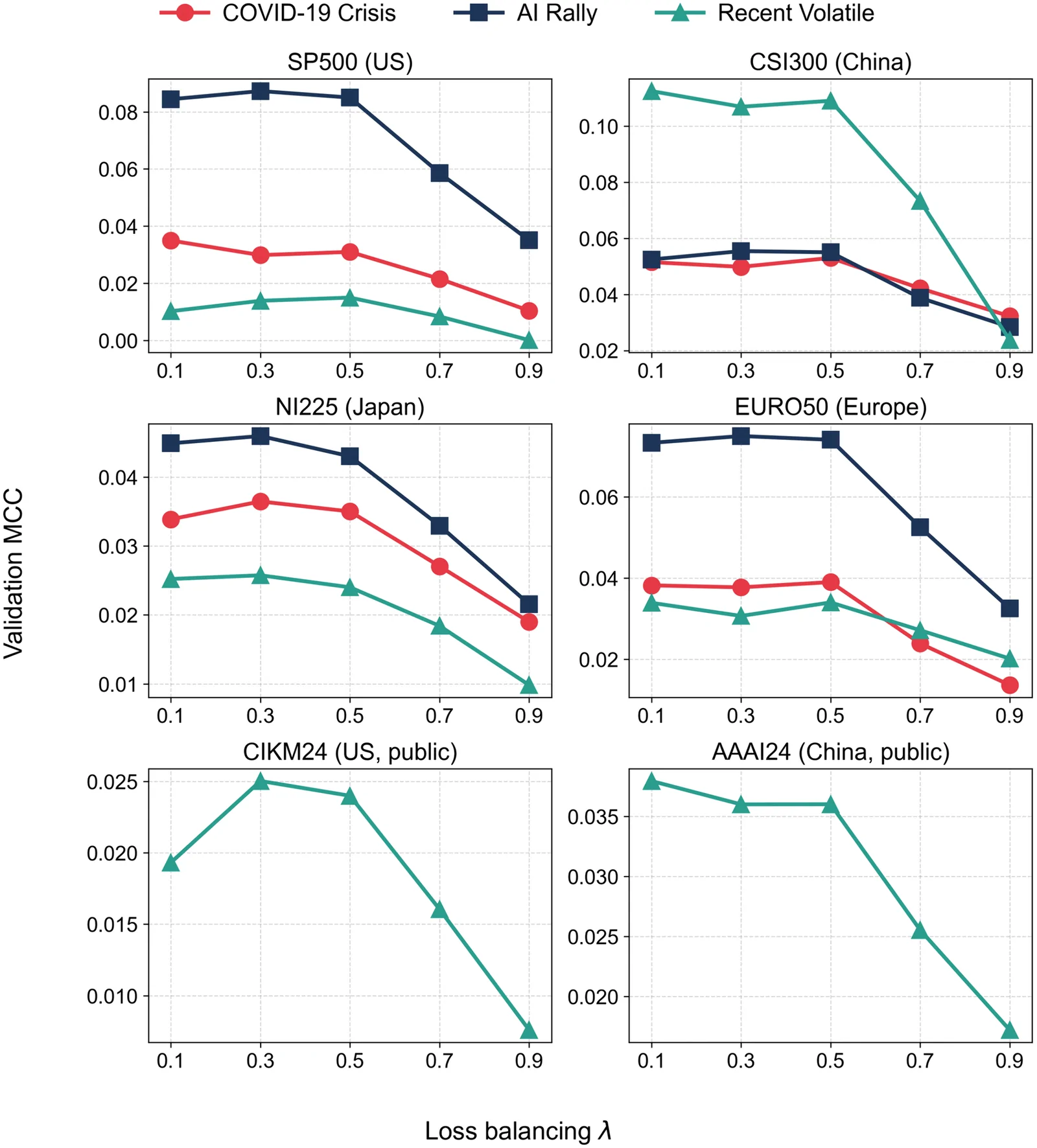
Validation-MCC sensitivity of craft to the loss balancing parameter λ.

**Fig 6:**
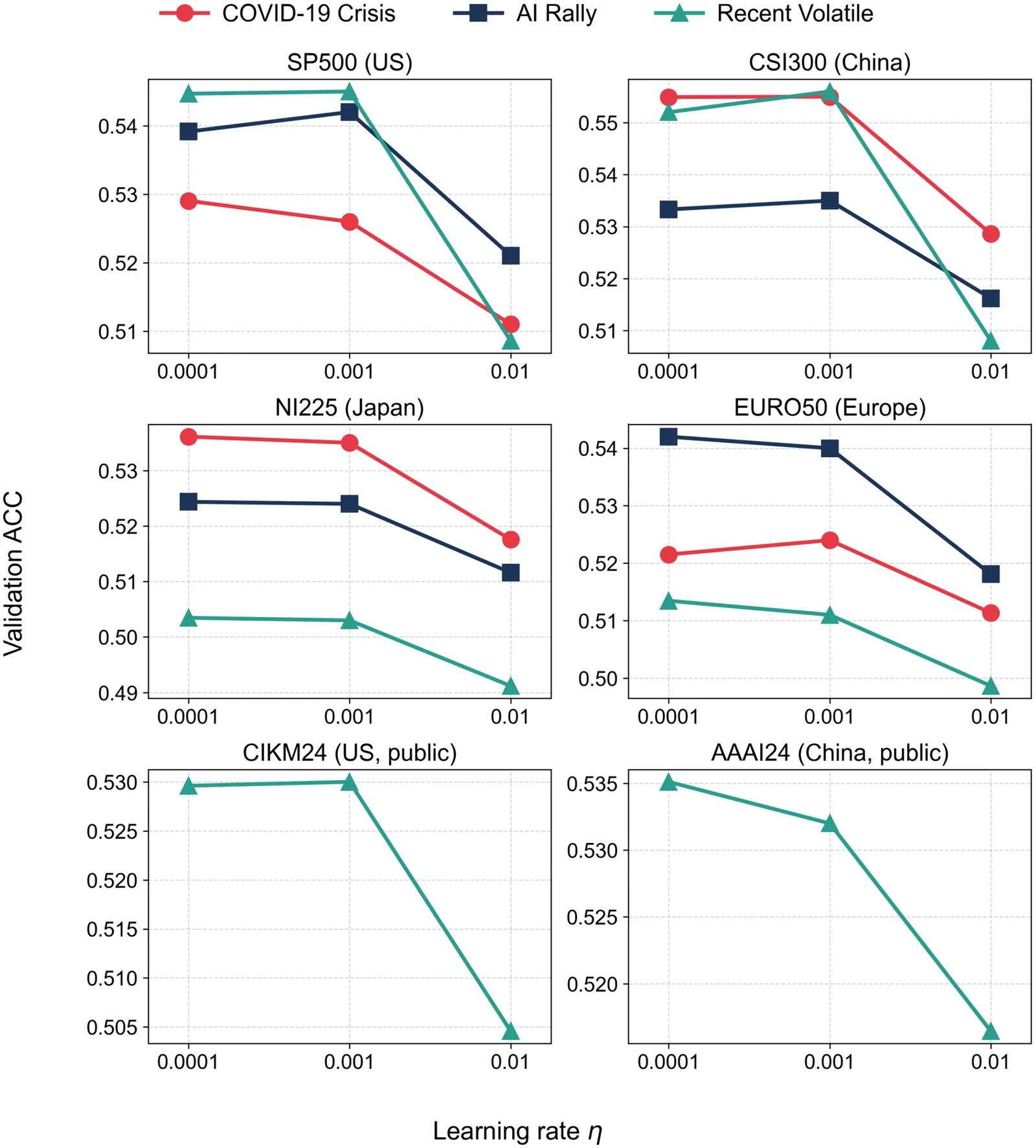
Validation-ACC sensitivity of craft to the learning rate η.

**Fig 7:**
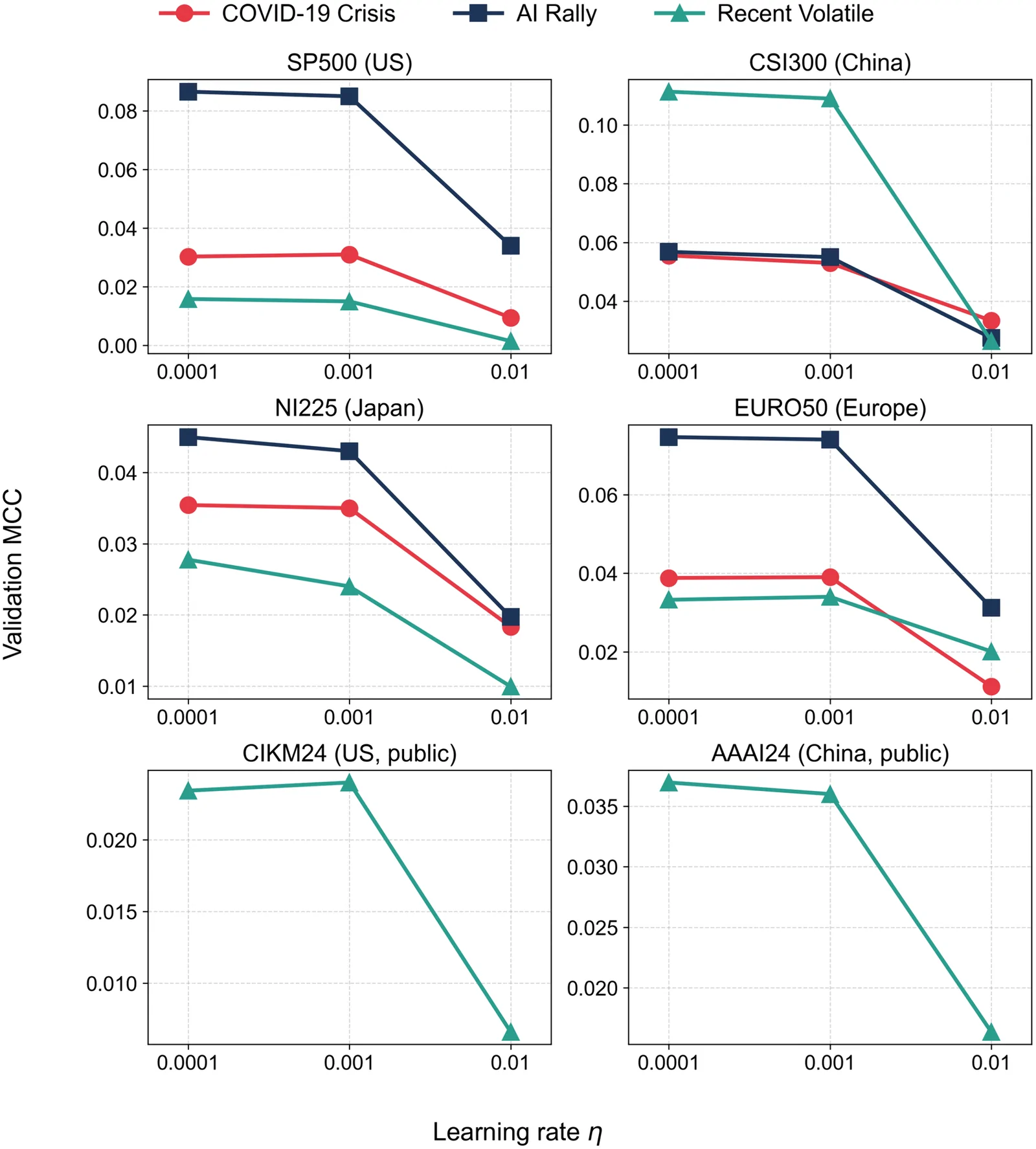
Validation-MCC sensitivity of craft to the learning rate η.

#### Implementation.

We train all models for 200 epochs on four NVIDIA GTX 1080 Ti GPUs (11 GB each), with an Intel Xeon Silver 4214 CPU and 503 GB of system memory. Table 3 summarizes Craft’s per-dataset compute budget. Trained in the same environment, MASTER is the lightest competitor at roughly 30–40 minutes per regime, whereas MATCC is comparable to or slightly heavier than Craft at roughly 2–3 hours. Craft’s training cost is therefore in line with existing market Transformers.

**Table 3:** Compute budget of Craft per dataset for 200-epoch training run on four GTX 1080 Ti GPUs. The *k*-means column is the centroid-construction time, which scales with the universe size. GPU-hours assume full utilization (wall time ×4). Latency (rebalance) is the end-to-end per-rebalance inference time over the full universe, and latency (asset) is the corresponding per-asset time.

Dataset	*k*-means	Training (200 ep)	GPU-hours	Peak GPU mem	Latency (rebalance)	Latency (asset)
SP500 (US)	19m 41s	3h 04m	12.3	9.1 GB	31.2 s	568 ms
CSI300 (China)	19m 12s	3h 01m	12.1	9.0 GB	29.6 s	539 ms
NI225 (Japan)	15m 33s	2h 51m	11.4	8.4 GB	25.8 s	587 ms
EURO50 (Europe)	13m 06s	2h 42m	10.8	7.9 GB	21.4 s	578 ms
CIKM24 (US)	18m 22s	3h 12m	12.8	8.8 GB	30.5 s	561 ms
AAAI24 (China)	17m 48s	3h 08m	12.5	8.6 GB	28.9 s	545 ms

### Stock price movement prediction performance (Q1)

Table 4 reports the fraction of up-days in each test period. Because the base rates stay near 0.50, an accuracy above 0.50 reflects genuine predictive signal rather than an exploitable label imbalance. The Chinese markets (CSI300, AAAI24) are mildly down-skewed, so a constant up-prediction would score below 0.50.

**Table 4:** Fraction of up-days (label *y* = 1) in each test period. Following the problem definition, a day is labeled up ($y_{i,t+1}=1$) when the next-day close exceeds the current close ($p_{i,t+1}>p_{i,t}$), and down (0) otherwise. Values lie near 0.50, indicating a nearly balanced task; the Chinese markets are mildly down-skewed.

Dataset	COVID-19 Crisis	AI Rally	Recent Volatile
SP500	0.49	0.53	0.52
CSI300	0.47	0.49	0.48
NI225	0.48	0.52	0.51
EURO50	0.48	0.52	0.51
CIKM24	—	—	0.52
AAAI24	—	—	0.48

S2 Table reports the full per-stock up-day rate distribution, whose medians likewise lie near 0.50.

We evaluate the predictive capabilities of Craft across three distinct market regimes to verify its robustness: the COVID-19 Crisis, the AI Rally, and the recent volatile period. Tables 5–7 report the three regimes in turn. Craft attains the highest ACC and MCC in every dataset and regime except the following. In the COVID-19 crisis, MASTER attains a higher ACC and MCC on CSI300. In the recent volatile period, FEDformer attains a higher ACC and MCC on NI225, and MATCC attains a higher MCC on AAAI24, while ALSTM matches rather than trails the MCC of Craft on CIKM24.

**Table 5:** Predictive performance (ACC and MCC) during the COVID-19 crisis period (2020-01-01 to 2020-03-31). Best results are in bold, and second-best are underlined. Val and Test denote the validation- and test-split scores. Test carries the ± standard deviation over five seeds, and Val is a point estimate. The classical baselines (Reversal, Momentum, ARMA, ARIMA-GARCH) are deterministic and reported from a single run.

Model	S&P500 (US)				CSI300 (China)			
	Val		Test		Val		Test	
	ACC (↑)	MCC (↑)	ACC (↑)	MCC (↑)	ACC (↑)	MCC (↑)	ACC (↑)	MCC (↑)
Reversal	0.483	−0.007	0.511	0.011	0.512	0.007	0.527	0.021
Momentum	0.491	−0.003	0.505	0.004	0.498	0.006	0.539	0.036
ARMA	0.508	−0.006	0.492	−0.011	0.520	0.021	0.533	0.043
ARIMA-GARCH	0.500	0.005	0.499	−0.003	0.501	0.018	0.520	0.029
LSTM	0.507	0.002	0.498 ± 0.004	0.002 ± 0.010	0.519	0.018	0.512 ± 0.005	0.015 ± 0.014
ALSTM	0.499	0.001	0.502 ± 0.003	0.005 ± 0.013	0.509	0.019	0.518 ± 0.008	0.021 ± 0.012
MA+LSTM	0.512	0.009	0.496 ± 0.007	0.009 ± 0.009	0.505	0.024	0.524 ± 0.010	0.035 ± 0.014
EMA+LSTM	0.510	0.002	0.513 ± 0.006	0.014 ± 0.011	0.520	0.025	0.534 ± 0.007	0.021 ± 0.015
ARIMA+LSTM	0.505	0.014	0.510 ± 0.004	0.003 ± 0.016	0.519	0.027	0.541 ± 0.009	0.041 ± 0.013
Wavelet+LSTM	0.497	0.007	0.506 ± 0.004	−0.011 ± 0.015	0.530	0.022	0.529 ± 0.008	0.046 ± 0.006
Kalman+LSTM	0.504	0.010	0.501 ± 0.004	−0.004 ± 0.013	0.532	0.023	0.538 ± 0.009	0.028 ± 0.013
PatchTST	0.502	0.006	0.507 ± 0.008	0.009 ± 0.009	0.518	0.020	0.521 ± 0.004	0.025 ± 0.010
Informer	0.514	0.009	0.498 ± 0.010	−0.001 ± 0.013	0.516	0.016	0.526 ± 0.008	0.034 ± 0.010
Autoformer	0.522	0.011	0.512 ± 0.006	0.014 ± 0.007	0.519	0.029	0.535 ± 0.003	0.045 ± 0.009
FEDformer	0.518	0.020	0.517 ± 0.007	0.019 ± 0.010	0.517	0.029	0.539 ± 0.007	0.039 ± 0.011
MATCC	0.522	0.017	0.516 ± 0.008	0.014 ± 0.011	0.539	0.036	0.533 ± 0.005	0.038 ± 0.014
MASTER	0.516	0.013	0.512 ± 0.005	0.011 ± 0.014	0.551	0.064	**0.552** ± 0.005	**0.062** ± 0.009
**CRAFT (proposed)**	0.526	0.031	**0.531** ± 0.006	**0.028** ± 0.009	0.555	0.053	0.545 ± 0.005	0.051 ± 0.012
**Model**	**NIKKEI225 (Japan)**				**EURO50 (Europe)**			
	Val		Test		Val		Test	
	ACC (↑)	MCC (↑)	ACC (↑)	MCC (↑)	ACC (↑)	MCC (↑)	ACC (↑)	MCC (↑)
Reversal	0.499	−0.004	0.510	−0.005	0.499	−0.005	0.506	−0.004
Momentum	0.503	−0.001	0.504	0.010	0.500	0.006	0.500	−0.012
ARMA	0.509	0.010	0.516	0.003	0.501	0.005	0.512	0.010
ARIMA-GARCH	0.513	0.013	0.497	0.017	0.514	0.005	0.493	0.003
LSTM	0.509	0.015	0.505 ± 0.006	0.008 ± 0.013	0.499	0.010	0.501 ± 0.005	0.004 ± 0.011
ALSTM	0.498	0.009	0.508 ± 0.008	0.012 ± 0.011	0.511	0.003	0.504 ± 0.004	0.006 ± 0.013
MA+LSTM	0.504	0.016	0.518 ± 0.002	0.015 ± 0.008	0.510	0.007	0.507 ± 0.006	0.002 ± 0.010
EMA+LSTM	0.508	0.013	0.511 ± 0.007	−0.005 ± 0.012	0.496	0.014	0.507 ± 0.005	−0.012 ± 0.008
ARIMA+LSTM	0.513	0.022	0.506 ± 0.004	0.009 ± 0.011	0.505	0.004	0.511 ± 0.007	−0.005 ± 0.009
Wavelet+LSTM	0.522	0.013	0.501 ± 0.007	0.002 ± 0.013	0.504	0.011	0.502 ± 0.006	0.008 ± 0.006
Kalman+LSTM	0.508	0.017	0.515 ± 0.004	0.020 ± 0.013	0.509	0.009	0.514 ± 0.005	0.013 ± 0.016
PatchTST	0.510	0.021	0.511 ± 0.004	0.015 ± 0.009	0.514	0.014	0.509 ± 0.007	0.008 ± 0.010
Informer	0.524	0.009	0.506 ± 0.004	0.005 ± 0.006	0.504	0.016	0.499 ± 0.008	0.002 ± 0.015
Autoformer	0.530	0.026	0.510 ± 0.010	0.019 ± 0.008	0.513	0.015	0.508 ± 0.008	0.011 ± 0.015
FEDformer	0.522	0.030	0.515 ± 0.003	0.025 ± 0.012	0.518	0.015	0.513 ± 0.010	0.018 ± 0.008
MATCC	0.517	0.019	0.522 ± 0.004	0.024 ± 0.008	0.529	0.023	0.518 ± 0.008	0.017 ± 0.011
MASTER	0.524	0.014	0.518 ± 0.005	0.021 ± 0.009	0.514	0.012	0.515 ± 0.003	0.013 ± 0.013
**CRAFT (proposed)**	0.535	0.035	**0.541** ± 0.008	**0.042** ± 0.014	0.524	0.039	**0.536** ± 0.007	**0.035** ± 0.011

**Table 6:** Predictive performance (ACC and MCC) during the AI rally period (2024-01-01 to 2024-06-30). Best results are in bold, and second-best are underlined. Val and Test denote the validation- and test-split scores. Test carries the ± standard deviation over five seeds, and Val is a point estimate. The classical baselines (Reversal, Momentum, ARMA, ARIMA-GARCH) are deterministic and reported from a single run.

Model	S&P500 (US)				CSI300 (China)			
	Val		Test		Val		Test	
	ACC (↑)	MCC (↑)	ACC (↑)	MCC (↑)	ACC (↑)	MCC (↑)	ACC (↑)	MCC (↑)
Reversal	0.503	0.011	0.500	0.032	0.498	0.009	0.496	0.023
Momentum	0.494	0.006	0.507	0.025	0.498	0.011	0.503	0.001
ARMA	0.500	0.013	0.519	0.010	0.508	0.010	0.515	0.009
ARIMA-GARCH	0.507	0.017	0.513	0.018	0.493	0.014	0.509	0.016
LSTM	0.520	0.015	0.508 ± 0.004	0.015 ± 0.009	0.511	0.008	0.504 ± 0.008	0.009 ± 0.008
ALSTM	0.520	0.017	0.512 ± 0.007	0.018 ± 0.013	0.504	0.011	0.507 ± 0.006	0.014 ± 0.010
MA+LSTM	0.510	0.013	0.518 ± 0.002	0.035 ± 0.011	0.505	0.006	0.510 ± 0.006	0.021 ± 0.011
EMA+LSTM	0.521	0.014	0.514 ± 0.005	0.024 ± 0.015	0.517	0.019	0.517 ± 0.010	0.001 ± 0.009
ARIMA+LSTM	0.509	0.025	0.521 ± 0.003	0.017 ± 0.008	0.520	0.009	0.505 ± 0.002	0.008 ± 0.012
Wavelet+LSTM	0.505	0.022	0.504 ± 0.005	0.030 ± 0.006	0.502	0.025	0.514 ± 0.009	0.026 ± 0.007
Kalman+LSTM	0.523	0.023	0.509 ± 0.009	0.010 ± 0.006	0.522	0.025	0.509 ± 0.010	0.015 ± 0.007
PatchTST	0.506	0.028	0.518 ± 0.006	0.024 ± 0.008	0.513	0.013	0.511 ± 0.005	0.019 ± 0.011
Informer	0.513	0.026	0.507 ± 0.006	0.023 ± 0.007	0.501	0.013	0.502 ± 0.008	0.010 ± 0.011
Autoformer	0.526	0.036	0.512 ± 0.008	0.035 ± 0.015	0.529	0.031	0.514 ± 0.007	0.017 ± 0.009
FEDformer	0.533	0.033	0.517 ± 0.007	0.040 ± 0.012	0.507	0.027	0.509 ± 0.003	0.025 ± 0.010
MATCC	0.520	0.039	0.525 ± 0.008	0.038 ± 0.010	0.524	0.026	0.521 ± 0.008	0.031 ± 0.010
MASTER	0.528	0.034	0.521 ± 0.004	0.032 ± 0.010	0.512	0.026	0.518 ± 0.004	0.028 ± 0.010
**CRAFT (proposed)**	0.542	0.085	**0.540** ± 0.005	**0.085** ± 0.013	0.535	0.055	**0.533** ± 0.004	**0.057** ± 0.014
**Model**	**NIKKEI225 (Japan)**				**EURO50 (Europe)**			
	Val		Test		Val		Test	
	ACC (↑)	MCC (↑)	ACC (↑)	MCC (↑)	ACC (↑)	MCC (↑)	ACC (↑)	MCC (↑)
Reversal	0.499	−0.008	0.506	0.010	0.501	0.004	0.512	0.013
Momentum	0.500	0.001	0.512	−0.005	0.500	0.014	0.518	0.027
ARMA	0.496	0.003	0.500	0.003	0.509	0.009	0.499	0.005
ARIMA-GARCH	0.515	0.010	0.493	0.017	0.512	0.013	0.506	0.020
LSTM	0.509	0.011	0.502 ± 0.006	0.005 ± 0.014	0.506	0.012	0.506 ± 0.008	0.012 ± 0.011
ALSTM	0.517	0.011	0.505 ± 0.007	0.008 ± 0.012	0.521	0.017	0.510 ± 0.004	0.015 ± 0.014
MA+LSTM	0.497	0.016	0.502 ± 0.004	0.002 ± 0.016	0.506	0.017	0.517 ± 0.009	0.030 ± 0.008
EMA+LSTM	0.512	0.005	0.514 ± 0.008	−0.005 ± 0.015	0.519	0.023	0.508 ± 0.005	0.005 ± 0.011
ARIMA+LSTM	0.514	0.019	0.511 ± 0.010	0.015 ± 0.010	0.513	0.022	0.513 ± 0.002	0.019 ± 0.014
Wavelet+LSTM	0.516	0.013	0.507 ± 0.009	0.009 ± 0.006	0.511	0.027	0.503 ± 0.006	0.025 ± 0.006
Kalman+LSTM	0.514	0.009	0.506 ± 0.007	0.020 ± 0.014	0.516	0.028	0.520 ± 0.004	0.012 ± 0.008
PatchTST	0.502	0.015	0.509 ± 0.003	0.014 ± 0.011	0.521	0.024	0.515 ± 0.004	0.021 ± 0.009
Informer	0.517	0.011	0.502 ± 0.006	0.002 ± 0.006	0.513	0.016	0.505 ± 0.005	0.012 ± 0.012
Autoformer	0.507	0.019	0.505 ± 0.009	0.015 ± 0.007	0.528	0.033	0.514 ± 0.009	0.022 ± 0.016
FEDformer	0.511	0.025	0.510 ± 0.009	0.021 ± 0.010	0.536	0.042	0.524 ± 0.009	0.035 ± 0.015
MATCC	0.506	0.023	0.518 ± 0.007	0.025 ± 0.009	0.529	0.034	0.522 ± 0.006	0.034 ± 0.011
MASTER	0.516	0.017	0.515 ± 0.003	0.022 ± 0.011	0.530	0.026	0.519 ± 0.008	0.030 ± 0.011
**CRAFT (proposed)**	0.524	0.043	**0.531** ± 0.006	**0.047** ± 0.010	0.540	0.074	**0.536** ± 0.007	**0.073** ± 0.011

**Table 7:** Predictive performance (ACC and MCC) during the recent volatile period. Best results are in bold, and second-best are underlined. Val and Test denote the validation- and test-split scores. Test carries the ± standard deviation over five seeds, and Val is a point estimate. The classical baselines (Reversal, Momentum, ARMA, ARIMA-GARCH) are deterministic and reported from a single run. Craft attains the highest accuracy on five of the six datasets (all except NI225), exceeding the best competitor by up to 4.1 ACC points and 7.8 MCC points.

Model	CIKM24 (US)				AAAI24 (China)				SP500 (US)			
	Val		Test		Val		Test		Val		Test	
	ACC	MCC	ACC	MCC	ACC	MCC	ACC	MCC	ACC	MCC	ACC	MCC
Reversal	0.512	−0.010	0.495	0.004	0.517	0.023	0.517	0.011	0.501	−0.010	0.506	−0.014
Momentum	0.513	0.007	0.508	−0.011	0.513	0.018	0.511	0.018	0.494	0.001	0.487	−0.001
ARMA	0.511	0.009	0.514	−0.003	0.532	0.025	0.498	0.003	0.486	0.008	0.500	−0.008
ARIMA-GARCH	0.518	0.012	0.502	0.011	0.517	0.027	0.505	0.025	0.500	0.000	0.494	−0.014
LSTM	0.518	0.006	0.509 ± 0.006	0.004 ± 0.014	0.529	0.034	0.521 ± 0.006	0.027 ± 0.010	0.489	−0.002	0.496 ± 0.006	0.004 ± 0.008
ALSTM	0.496	0.013	0.499 ± 0.003	**0.019** ± 0.012	0.512	0.011	0.519 ± 0.003	0.018 ± 0.014	0.485	0.003	0.493 ± 0.007	0.006 ± 0.012
MA+LSTM	0.509	0.010	0.516 ± 0.004	0.003 ± 0.009	0.516	0.026	0.523 ± 0.008	0.003 ± 0.014	0.498	0.007	0.496 ± 0.004	−0.009 ± 0.011
EMA+LSTM	0.520	0.004	0.514 ± 0.005	0.014 ± 0.008	0.527	0.037	0.512 ± 0.008	0.017 ± 0.007	0.497	0.011	0.501 ± 0.008	0.002 ± 0.006
ARIMA+LSTM	0.520	0.011	0.499 ± 0.008	−0.011 ± 0.006	0.513	0.027	0.507 ± 0.002	0.023 ± 0.010	0.494	0.009	0.508 ± 0.009	−0.014 ± 0.016
Wavelet+LSTM	0.524	0.010	0.513 ± 0.004	0.009 ± 0.013	0.529	0.033	0.519 ± 0.002	0.010 ± 0.015	0.511	0.001	0.505 ± 0.008	−0.014 ± 0.015
Kalman+LSTM	0.519	0.011	0.520 ± 0.008	−0.004 ± 0.012	0.516	0.027	0.502 ± 0.009	0.028 ± 0.012	0.512	0.005	0.501 ± 0.009	−0.003 ± 0.007
PatchTST	0.486	−0.012	0.486 ± 0.003	−0.009 ± 0.014	0.516	0.024	0.513 ± 0.008	0.029 ± 0.012	0.506	0.001	0.494 ± 0.008	−0.002 ± 0.012
Informer	0.492	0.002	0.497 ± 0.007	0.000 ± 0.006	0.512	0.024	0.502 ± 0.003	0.013 ± 0.010	0.499	0.002	0.492 ± 0.010	−0.010 ± 0.008
Autoformer	0.495	0.001	0.509 ± 0.007	0.009 ± 0.008	0.509	0.023	0.509 ± 0.004	0.022 ± 0.016	0.514	0.011	0.504 ± 0.009	0.000 ± 0.011
FEDformer	0.514	0.015	0.512 ± 0.008	0.013 ± 0.009	0.511	0.034	0.516 ± 0.006	0.025 ± 0.006	0.494	0.008	0.502 ± 0.004	0.007 ± 0.012
MATCC	0.504	0.012	0.503 ± 0.003	0.006 ± 0.008	0.510	0.046	0.521 ± 0.008	**0.046** ± 0.010	0.512	−0.003	0.501 ± 0.003	0.003 ± 0.012
MASTER	0.491	−0.017	0.490 ± 0.006	−0.010 ± 0.014	0.525	0.052	0.518 ± 0.004	0.045 ± 0.010	0.502	−0.023	0.512 ± 0.004	−0.017 ± 0.010
**CRAFT (proposed)**	0.530	0.024	**0.531** ± 0.008	**0.019** ± 0.010	0.532	0.036	**0.526** ± 0.003	0.033 ± 0.008	0.545	0.015	**0.539** ± 0.006	**0.009** ± 0.014
**Model**	**CSI300 (China)**				**NI225 (Japan)**				**EURO50 (Europe)**			
	Val		Test		Val		Test		Val		Test	
	ACC	MCC	ACC	MCC	ACC	MCC	ACC	MCC	ACC	MCC	ACC	MCC
Reversal	0.493	0.003	0.490	0.018	0.503	0.008	0.485	0.015	0.505	0.018	0.508	0.014
Momentum	0.497	0.002	0.509	−0.004	0.504	0.013	0.489	0.008	0.513	0.012	0.514	0.007
ARMA	0.511	0.006	0.497	0.004	0.496	0.022	0.501	−0.007	0.500	0.011	0.502	0.021
ARIMA-GARCH	0.515	0.001	0.503	0.011	0.513	0.024	0.495	0.001	0.506	0.023	0.495	−0.001
LSTM	0.506	0.009	0.504 ± 0.004	0.003 ± 0.008	0.502	0.016	0.505 ± 0.005	0.016 ± 0.008	0.498	0.022	0.509 ± 0.006	0.018 ± 0.012
ALSTM	0.502	0.020	0.507 ± 0.003	0.026 ± 0.010	0.496	0.021	0.507 ± 0.006	0.019 ± 0.009	0.510	0.004	0.499 ± 0.005	−0.001 ± 0.014
MA+LSTM	0.518	0.008	0.508 ± 0.008	0.010 ± 0.006	0.501	0.014	0.496 ± 0.008	0.018 ± 0.009	0.510	0.021	0.516 ± 0.005	0.013 ± 0.013
EMA+LSTM	0.508	0.002	0.510 ± 0.005	0.016 ± 0.010	0.509	0.017	0.506 ± 0.004	−0.007 ± 0.014	0.504	0.016	0.511 ± 0.010	0.006 ± 0.006
ARIMA+LSTM	0.516	0.005	0.511 ± 0.007	0.003 ± 0.010	0.511	0.017	0.491 ± 0.009	0.007 ± 0.006	0.526	0.028	0.509 ± 0.007	0.024 ± 0.006
Wavelet+LSTM	0.518	0.007	0.494 ± 0.007	−0.004 ± 0.013	0.496	0.019	0.500 ± 0.008	−0.000 ± 0.015	0.527	0.034	0.513 ± 0.004	−0.001 ± 0.013
Kalman+LSTM	0.503	0.017	0.515 ± 0.006	0.021 ± 0.015	0.514	0.017	0.508 ± 0.009	0.013 ± 0.010	0.530	0.024	0.520 ± 0.007	0.029 ± 0.009
PatchTST	0.503	0.012	0.495 ± 0.008	0.005 ± 0.014	0.509	0.000	0.498 ± 0.005	−0.004 ± 0.010	0.506	−0.005	0.496 ± 0.004	−0.003 ± 0.009
Informer	0.490	0.007	0.497 ± 0.010	0.009 ± 0.010	0.489	0.006	0.493 ± 0.005	0.003 ± 0.016	0.513	0.005	0.500 ± 0.003	0.009 ± 0.009
Autoformer	0.516	0.017	0.504 ± 0.005	0.016 ± 0.014	0.509	0.002	0.496 ± 0.006	0.018 ± 0.011	0.520	0.015	0.515 ± 0.006	0.016 ± 0.014
FEDformer	0.517	0.016	0.510 ± 0.010	0.020 ± 0.009	0.511	0.019	**0.514** ± 0.007	**0.027** ± 0.015	0.521	0.022	0.507 ± 0.003	0.021 ± 0.014
MATCC	0.514	0.016	0.511 ± 0.004	0.021 ± 0.014	0.501	0.011	0.504 ± 0.006	0.010 ± 0.010	0.506	0.029	0.515 ± 0.005	0.026 ± 0.012
MASTER	0.495	0.014	0.503 ± 0.003	0.010 ± 0.010	0.496	−0.005	0.499 ± 0.004	−0.001 ± 0.013	0.497	0.010	0.503 ± 0.006	0.007 ± 0.012
**CRAFT (proposed)**	0.556	0.109	**0.556** ± 0.004	**0.104** ± 0.009	0.503	0.024	0.509 ± 0.006	0.023 ± 0.008	0.511	0.034	**0.523** ± 0.008	**0.037** ± 0.011

By effectively smoothing high-frequency noise through centroid-based tokenization and capturing dynamic inter-stock correlations, our model maintains robust predictive power regardless of extreme macroeconomic shifts.

#### 1) COVID-19 crisis period (2020−01 ~ 2020−03).

We analyze performance during the extreme volatility of the pandemic onset. Table 5 summarizes the results. In this panic-driven market, Craft generally demonstrates superior stability, achieving the highest ACC and MCC in the US, Japan, and Europe. Notably, in the Chinese market (CSI300), MASTER achieves a slightly higher ACC (0.552) than Craft (0.545). This suggests that MASTER’s formulation aggressively fits the specific policy-driven dynamics of the Chinese market during this anomaly. However, Craft outperforms MASTER in all other regions, confirming that our approach effectively suppresses high-frequency noise to maintain robust performance during global turbulence.

#### 2) AI rally period (2024−01 ~ 2024−06).

Next, we examine the performance during the strong bullish trend driven by the technology sector (Table 6). In this regime, Craft consistently achieves the highest ACC and MCC across all evaluated datasets. By effectively capturing the global context of tech-driven momentum via dynamic stock embeddings, Craft successfully identifies and leverages the upward trends better than competitors.

#### 3) Recent volatile period.

Finally, Table 7 presents the comprehensive performance on all six datasets covering the full test periods, which natively embody recent volatile market conditions. Craft achieves the highest ACC on all evaluated benchmarks except NI225, where FEDformer’s moving-average series decomposition transfers better to this market. Unlike MATCC, which uses a RWKV-based model, Craft combines a token-based Transformer decoder with a dynamically weighted related-stock context. This architecture provides stronger robustness against random spikes, resulting in superior ACC and MCC across most datasets. Because the market-feature block in MASTER and MATCC is engineered for market-specific dynamics, its contribution varies across markets. This variation is the largest for MASTER on the US benchmarks (CIKM24 and SP500), where its MCC drops to near or slightly below zero. Among the general-purpose Transformers, Autoformer and FEDformer apply moving-average series decomposition, which suppresses part of the high-frequency noise and places them above Informer. Because equity prices exhibit weak and unstable seasonality [45], this periodic decomposition transfers only partially to daily movement prediction. Among the preprocessing baselines, the adaptive smoothers EMA+LSTM and Kalman+LSTM improve over a plain LSTM and reach the level of the attention-based ALSTM on most datasets, whereas the simpler moving-average and wavelet variants are more uneven. This confirms that denoising the raw prices yields a measurable but limited benefit, which Craft’s centroid tokenization consistently surpasses. On CIKM24, the MCC of Craft (0.019) ties that of ALSTM, while Craft leads ALSTM by 3.2%p in ACC (0.531 vs. 0.499). We assess the statistical significance of these prediction differences later in this section.

We also investigate the impact of the number *k* of centroids on model performance. Fig 8 reports the validation-ACC sensitivity to *k*, selected independently for each market regime on that regime’s validation split. Since the criterion is downstream validation accuracy, *k* is task-supervised rather than purely unsupervised. The per-dataset optima are spread across the grid, but the cross-dataset mean peaks 1) at *k* = 200 for the COVID-19 crisis and recent volatile regimes, and 2) at *k* = 150 for the AI rally regime.

**Fig 8:**
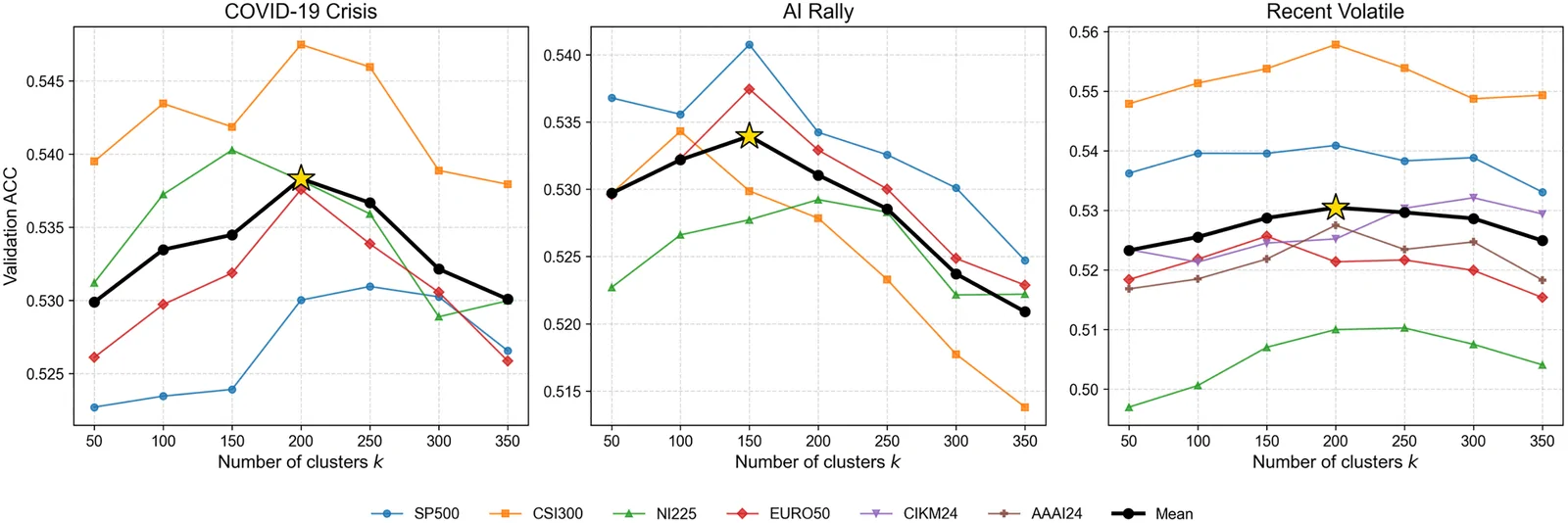
*k*-means cluster-count sensitivity, shown separately for the three market regimes. Within each regime, colored curves are the per-dataset validation accuracy and the black curve is the cross-dataset mean. The star marks the *k* selected on each regime’s validation split: *k* = 200 for the COVID-19 crisis and recent volatile regimes, and *k* = 150 for the AI rally regime. Each curve is averaged over five random seeds. The per-regime validation windows are given in Table 2.

Lastly, Fig 9 highlights distinct token-label distributions per market. The Chinese market (CSI300) has a small number of dominant tokens, covering a significant portion of the observations. We observe that Craft naturally adapts to such highly skewed token distributions, leading to the observed regional differences in performance.

**Fig 9:**
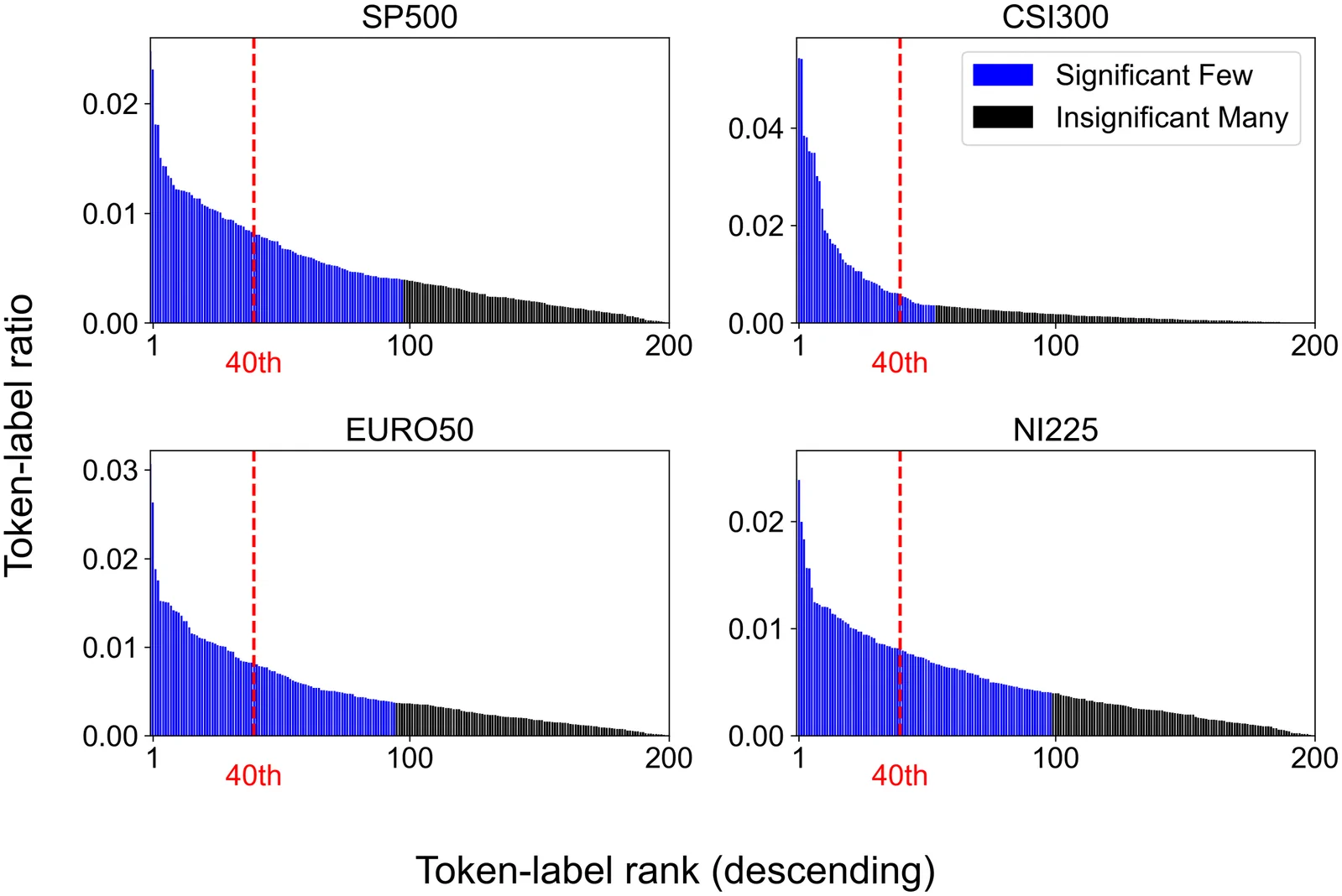
Token-label ratio distribution across the four new datasets (SP500, CSI300, NI225, EURO50). Different markets exhibit varying token-label distributions, contributing to observed performance differences.

The ACC values in Tables 5–7 lie in the 0.50–0.56 range, and the MCC values remain close to zero. These magnitudes are typical of daily stock-direction prediction, where small accuracy gains over 0.50 baseline translate into materially different investment outcomes.

### Investment simulation (Q2)

To translate our predictive performance into real-world profitability, we simulate investment strategies across the three regimes under realistic trading frictions. We charge a one-way transaction cost of 6 bps on each sell, and to protect profitability under this cost we gate each position by 60 % probability threshold that limits turnover. At the close of day *t*, a model takes a long position in stock *i* for day *t* + 1 only when its predicted $p({\hat{y}}_{i,t+1}=1)$ exceeds 0.60, and analogously a short position when $p({\hat{y}}_{i,t+1}=0)$ exceeds 0.60. Eligible longs and shorts are equal-weighted within each leg. Each position is entered at the closing price of day *t* and unwound at the closing price of day *t* + 1, earning the close-to-close return. For CSI300, where unconstrained shor*t*ing of the full A-share universe is not realis*t*ic, we disable the short leg (long-only). Because the resulting long-short portfolio is cash-neutral, the ASR is computed against a zero risk-free rate. Entering at the same day-*t* close from which the signal is computed is an idealized fill that ignores intraday execution latency, bu*t* this protocol is applied identically to every competing method, so the relative comparison among methods remains fair. As a robustness check, we recompute the annualized Sharpe ratio of every learned method under a stricter two-sided cost that also charges 6 bps on buys, over the recent volatile period. Craft retains the highest value on four of the six datasets (S3 Table).

Fig 10 reports the resulting portfolio-value trajectories for all six datasets. Flat segments correspond to periods during which a model’s predicted probabilities do not exceed the 0.60 threshold, so no positions are opened. Craft attains the highest cumulative return on five of the six datasets. For instance, on CIKM24 Craft delivers +98% cumulative return against the Market’s +85%, and on S&P500 + 33% return against the Market’s +26%. NIKKEI225 is the sole exception, where FEDformer’s series decomposition narrowly leads.

**Fig 10:**
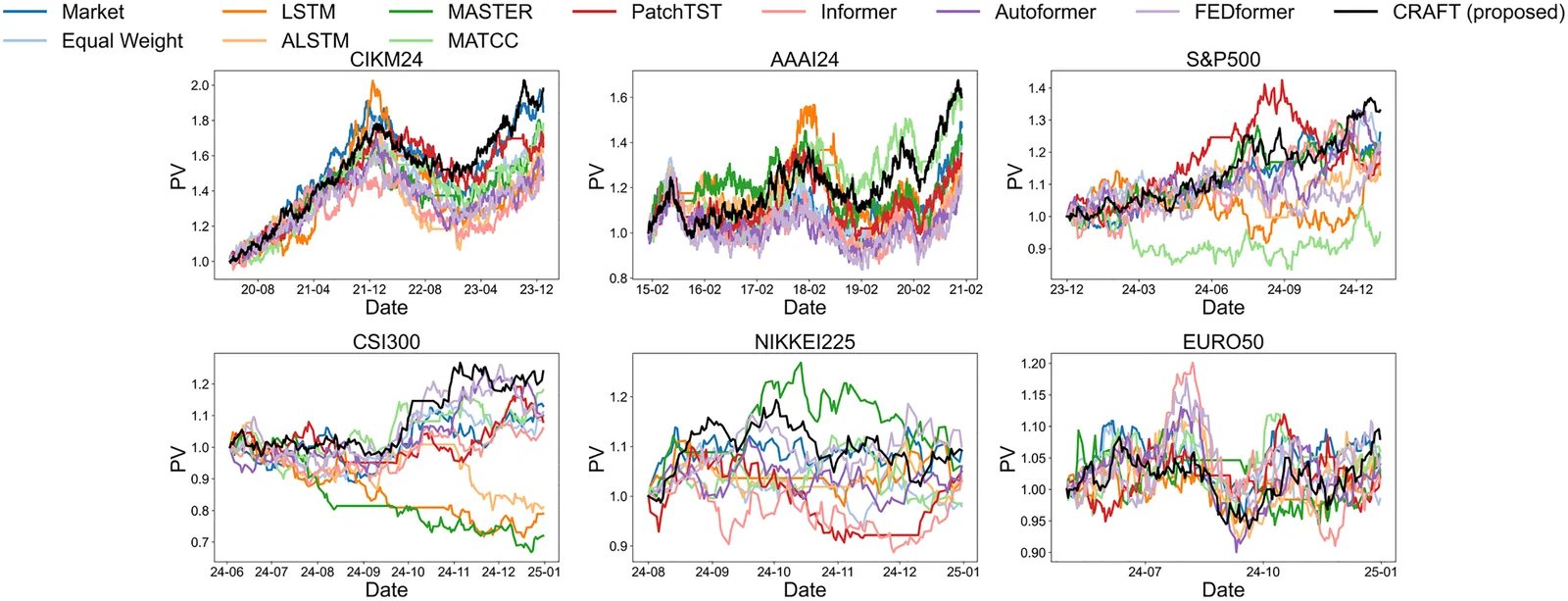
Cumulative portfolio-value trajectories of Craft (proposed) and competing baselines across the six datasets. Positions follow the 60 % probability-threshold gated long-short strategy described in the text. Craft (proposed) is shown in black. Flat segments indicate windows during which the model’s top-class probability does not exceed the threshold, so no positions are opened. The short leg is disabled for CSI300 to respect A-share short-selling constraints.

#### 1) COVID-19 crisis period.

Table 8 reports the performance during the 2020 market crash. While MASTER achieves a higher ASR exclusively in the Chinese market (1.551 vs. Craft’s 1.342), Craft consistently demonstrates vastly superior stability by maintaining significantly lower RMDD across all evaluated markets. For instance, on the S&P500 dataset, Craft reduces the maximum drawdown to 18.2%, compared to MASTER’s 35.1%. This superior risk management is inherently driven by Craft’s stock-axis attention network, which effectively captures flight-to-safety dynamics and executes conservative strategies during extreme turbulence.

**Table 8:** Investment simulation results (ASR and RMDD) during the COVID-19 Crisis period (2020-01-01 to 2020-03-31). Best results are in bold, and second-best are underlined.

Model	S&P500 (US)		CSI300 (China)	
	ASR (↑)	RMDD (↓)	ASR (↑)	RMDD (↓)
LSTM	−0.521	41.2	−0.893	26.3
ALSTM	−1.145	48.5	−0.652	23.8
PatchTST	−0.952	45.3	−1.124	28.5
Informer	−0.873	44.1	−0.948	27.1
Autoformer	−0.640	39.8	−0.731	24.6
FEDformer	−0.508	38.2	−0.594	22.9
MATCC	−0.453	30.5	−0.151	20.5
MASTER	1.452	35.1	**1.551**	22.4
**CRAFT (proposed)**	**1.954**	**18.2**	1.342	**14.2**
**Model**	**NIKKEI225 (Japan)**		**EURO50 (Europe)**	
	ASR (↑)	RMDD (↓)	ASR (↑)	RMDD (↓)
LSTM	−0.684	35.1	−1.251	38.4
ALSTM	−0.842	40.5	−1.624	42.1
PatchTST	−1.053	38.2	−1.552	40.3
Informer	−0.776	37.4	−1.318	39.1
Autoformer	−0.523	33.6	−1.042	35.7
FEDformer	−0.402	31.8	−0.889	34.2
MATCC	−0.421	28.5	−0.654	30.2
MASTER	0.852	29.8	1.152	33.5
**CRAFT (proposed)**	**1.254**	**16.5**	**1.503**	**17.8**

#### 2) AI rally period.

During the recent bullish trend (Table 9), Craft consistently achieves the highest ASR and the lowest RMDD across all datasets. This confirms that our model successfully capitalizes on strong sectoral momentum.

**Table 9:** Investment simulation results (ASR and RMDD) during the AI Rally period (2024-01-01 to 2024-06-30). Best results are in bold, and second-best are underlined.

Model	S&P500 (US)		CSI300 (China)	
	ASR (↑)	RMDD (↓)	ASR (↑)	RMDD (↓)
LSTM	0.852	18.4	0.651	16.2
ALSTM	0.924	16.5	0.725	15.5
PatchTST	1.152	14.2	0.453	18.5
Informer	0.806	17.9	0.588	17.2
Autoformer	0.948	15.8	0.704	15.9
FEDformer	1.043	15.1	0.769	15.3
MATCC	1.254	10.5	0.852	13.5
MASTER	1.651	12.4	1.154	14.2
**CRAFT (proposed)**	**2.842**	**6.8**	**1.653**	**8.5**
**Model**	**NIKKEI225 (Japan)**		**EURO50 (Europe)**	
	ASR (↑)	RMDD (↓)	ASR (↑)	RMDD (↓)
LSTM	0.552	15.8	0.821	14.5
ALSTM	0.624	14.5	0.952	13.8
PatchTST	0.851	12.4	1.054	12.5
Informer	0.519	15.1	0.788	14.1
Autoformer	0.642	13.9	0.916	12.9
FEDformer	0.728	13.2	1.007	12.1
MATCC	0.953	10.8	1.152	11.2
MASTER	1.052	11.5	1.354	10.5
**CRAFT (proposed)**	**1.524**	**7.2**	**1.851**	**6.5**

#### 3) Recent volatile period.

Table 10 displays the results for the full test period. Craft achieves the highest ASR on five of the six datasets, with NI225 the sole exception, and outperforms the best competitor by up to 1.0. Relative to the best alternative, Craft reduces RMDD by up to 7.0% points, ensuring robust returns even in volatile markets.

**Table 10:** Investment simulation: Annualized Sharpe Ratio (ASR) and Relative Maximum Drawdown (RMDD, %) across all six datasets. Best performance per column in bold, and second-best are underlined.

	CIKM24 (US)		AAAI24 (China)		S&P500 (US)	
Method	ASR (↑)	RMDD (↓)	ASR (↑)	RMDD (↓)	ASR (↑)	RMDD (↓)
Equal Weight	0.622	20.0	0.417	22.0	1.089	9.2
Buy-and-Hold	0.450	24.0	0.280	26.0	0.850	11.5
LSTM	0.812	15.8	0.845	18.5	1.032	6.7
ALSTM	0.645	18.2	0.612	21.4	1.664	6.4
PatchTST	0.125	24.5	0.315	25.6	1.219	6.7
Informer	0.582	19.2	0.608	21.0	0.981	7.4
Autoformer	0.689	17.6	0.703	19.5	1.146	7.1
FEDformer	0.741	16.9	0.781	18.9	1.283	6.9
MASTER	0.245	22.1	0.712	20.1	0.789	7.2
MATCC	0.753	16.5	0.952	17.8	−0.242	**5.8**
**CRAFT (proposed)**	**1.412**	**11.2**	**1.215**	**14.5**	**2.334**	6.1
	**CSI300 (China)**		**NIKKEI225 (Japan)**		**EURO50 (Europe)**	
**Method**	ASR (↑)	RMDD (↓)	ASR (↑)	RMDD (↓)	ASR (↑)	RMDD (↓)
Equal Weight	0.263	14.4	−0.144	19.8	−0.103	10.2
Buy-and-Hold	0.150	17.0	−0.250	23.0	−0.200	12.5
LSTM	−1.427	19.6	0.158	13.4	0.370	6.7
ALSTM	−1.347	19.6	0.123	16.4	0.371	6.8
PatchTST	0.003	11.4	0.194	10.9	0.221	6.3
Informer	0.061	13.2	0.089	14.7	0.188	8.1
Autoformer	0.108	12.6	0.207	13.8	0.276	7.7
FEDformer	0.142	12.1	**0.491**	12.8	0.338	7.3
MASTER	−1.914	14.6	−0.038	16.5	0.165	7.9
MATCC	0.167	11.0	−0.106	**9.6**	0.289	7.5
**CRAFT (proposed)**	**1.267**	**4.0**	0.421	11.2	**1.351**	**4.6**

Table 11 reports, per dataset and regime, the Diebold–Mariano (DM) test on daily loss differentials, the Pesaran–Timmermann (PT) test of Craft’s directional skill, and the Jobson–Korkie test with Memmel correction on the Sharpe-ratio difference, each comparing Craft against the strongest baseline. The Diebold–Mariano test uses the 0–1 directional loss aggregated across listed stocks into one value per trading day, with a Newey–West HAC variance at truncation lag $\lfloor 1.5\;T^{1/3}\rfloor$. The Pesaran–Timmermann test pools all (stock, day) pairs. The reported *p*-values are per dataset–regime cell, without adjustment for multiplicity across cells. The DM and PT tests are each significant on 10 of the 14 dataset–regime pairs. The four DM exceptions are the two splits where Craft trails the strongest baseline (CSI300 in the COVID-19 crisis and NI225 in the recent volatile period) and the two where Craft leads by only 0.3%p in accuracy (AAAI24 and EURO50 in the recent volatile period). The JK test reaches significance mainly on the recent-volatile datasets (four of six). The COVID-19 and AI-rally windows are shorter (about three and six months), so their smaller samples rarely yield significant Sharpe-ratio differences.

**Table 11:** Statistical significance of Craft against the strongest baseline on each dataset and regime. DM: Diebold–Mariano test on daily loss differentials (HAC-robust). PT: Pesaran–Timmermann test of Craft’s directional skill. JK: Jobson–Korkie test with Memmel correction on the Sharpe-ratio difference. Entries are *p*-values; ${\;}^{*}p<0.05$, ${\;}^{**}p<0.01$, ${\;}^{***}p<0.001$; a value without a star is not significant (p≥0.05).

Regime	Dataset	DM (loss)	PT (direction)	JK (Sharpe)
COVID-19 crisis	SP500	0.012^*^	0.021^*^	0.17
	CSI300	0.58	0.034^*^	0.49
	NI225	0.007^**^	0.022^*^	0.23
	EURO50	0.009^**^	0.030^*^	0.27
AI rally	SP500	0.008^**^	0.048^*^	0.041^*^
	CSI300	0.024^*^	0.026^*^	0.13
	NI225	0.022^*^	0.057	0.16
	EURO50	0.031^*^	0.029^*^	0.12
Recent volatile	CIKM24	0.033^*^	0.030^*^	0.044^*^
	AAAI24	0.27	0.061	0.22
	SP500	0.001^***^	0.013^*^	0.025^*^
	CSI300	<0.001^***^	<0.001^***^	0.008^**^
	NI225	0.41	0.19	0.21
	EURO50	0.22	0.073	0.021^*^

### Stock embedding quality (Q3)

Craft leverages the dynamic global context of each stock by applying SVD to the correlation matrix at every time step. Specifically, at time *t*, Craft constructs a correlation matrix from the past 60 days of returns for all individual stocks, and then computes each stock’s embedding via SVD. These embeddings incorporate *temporal* information, since the pairwise correlation among stocks varies over time.

Table 12 shows the five closest neighbours from the dynamic embeddings for TSLA (Tesla) and BAC (Bank of America) at two snapshots (2021 and 2024). We specifically select these two stocks to demonstrate the model’s capacity to capture contrasting temporal dynamics: TSLA represents a case where market correlations shift dramatically over time, while BAC illustrates a highly stable sectoral relationship. In 2021 TSLA is nearest to Big–Tech stocks such as AAPL and AVGO. By late 2024 its closest neighbours shift to AI–focused firms like NVDA and MSFT. This shift illustrates how the embeddings track the market’s AI boom. BAC stays close to JPM and WFC at both times, confirming a stable financial–sector relationship that our method captures over time.

**Table 12:** Top-5 most similar stocks (by Euclidean distance) returned by our dynamic embeddings for four query snapshots. For TSLA on 2024-12-31, the global context most strongly references the prices of NVDA, AVGO, AAPL, MSFT, and GOOGL.

Query	Top 1	Top 2	Top 3	Top 4	Top 5
TSLA @ 2021-06-28	AAPL	AVGO	MA	META	ECL
TSLA @ 2024-12-31	NVDA	AVGO	AAPL	MSFT	GOOGL
BAC @ 2021-06-28	JPM	WELL	WFC	DUK	MCD
BAC @ 2024-12-31	WFC	JPM	ECL	LIN	APD

Craft effectively captures the context of stocks that share strong correlations or exhibit similar characteristics, since it forms each stock’s global context by taking a weighted average of embeddings based on pairwise distances. This mechanism enables Craft to reflect meaningful interdependencies among stocks and provides a robust global perspective for subsequent predictions.

### Ablation study (Q4)

Fig 11 compares Craft with its ablated variants.

**Fig 11:**
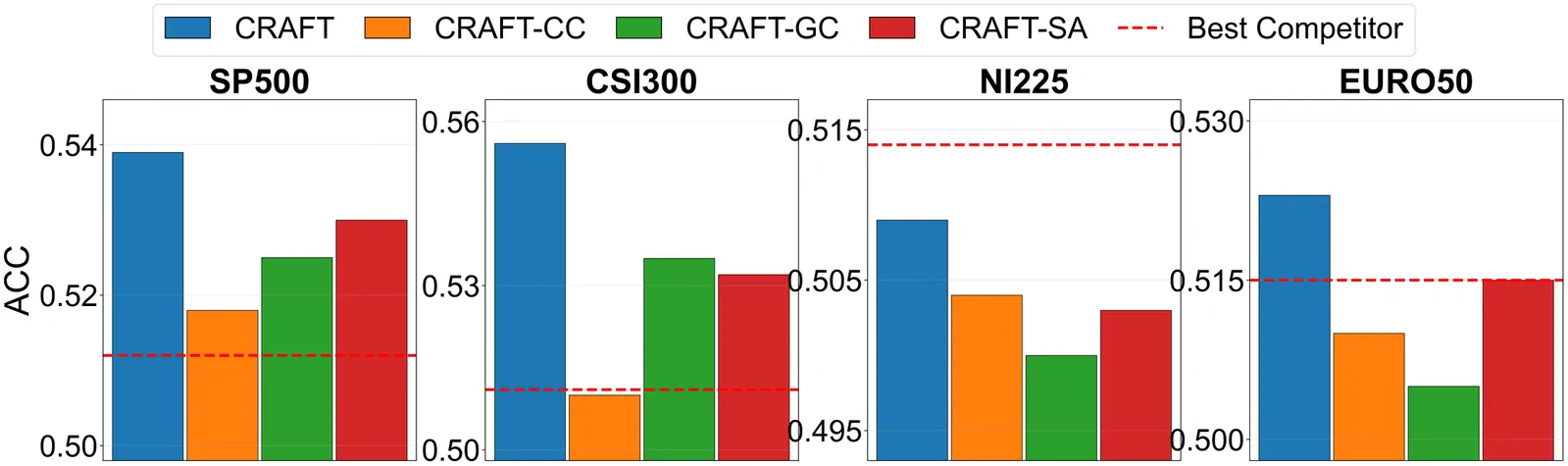
Ablation study of Craft on the four new datasets (SP500, CSI300, NI225, EURO50). The dashed line indicates the best competitor on each dataset. Removing the centroid-based clustering (-CC) causes the largest accuracy drop in all four markets.

- **CRAFT-CC**: Craft *without* centroid-based clustering (CC) module.
- **CRAFT-GC**: Craft *without* global context (GC) feature.
- **CRAFT-SA**: Craft *without* stock-axis attention (SA) module.

We observe that each omitted module leads to a drop in prediction accuracy, and the full Craft achieves the best performance. This indicates that the centroid–based clustering reduces inherent randomness, the global context leverages correlated stock patterns, and the inter-stock glocal context enhances the overall prediction accuracy.

## Conclusion

We propose Craft, an accurate method for stock price movement prediction that attentively integrates *glocal* patterns from multiple stocks through both time-axis and stock-axis contexts, without requiring prior knowledge. Craft operates in three key phases: 1) converting stock prices into centroid-based tokens to mitigate inherent randomness, 2) generating a multi-level context that combines local token sequences with a dynamic global context via correlation-based embeddings, and 3) unifying time-axis and stock-axis attention in an end-to-end framework, maximizing predictive performance through coordinated learning of local and inter-stock patterns. Experiments on datasets from major world markets show that Craft achieves the highest accuracy in twelve of the fourteen test settings and ranks the second in the other two, improving accuracy by up to 4.1%p and the Matthews correlation coefficient by up to 7.8 points over the best competitors. In addition, Craft improves the annualized Sharpe ratio by up to 1.0 and reduces the maximum drawdown by up to 7.0%p over the best competitor. The learned global context embeddings also provide new perspectives on how stocks interrelate in both local and global domains.

## Supporting information

S1 Table Measured final-epoch training-loss magnitudes of Craft. (PDF)

S2 Table Per-stock up-day rate distribution over the recent-volatile test window. (PDF)

S3 Table Investment performance under a two-sided transaction cost. (PDF)
