# Supplementary material for: Accurate stock movement prediction via centroid-based randomness smoothing
Source: PLoS One. 2026 Oct 8;21(10):e0345252. doi: 10.1371/journal.pone.0345252 (PMC13649149; doi:10.1371/journal.pone.0345252)
Supplement: Supplementary file 1 [file pone.0345252.s001.pdf]

S1 Table. Measured final-epoch training-loss magnitudes of Craft. Final-epoch magnitudes of the two loss terms, read directly from the training logs, for the recent-volatile regime and averaged over five random seeds.

The centroid tokenization loss L_{time} (a 200-class cross-entropy) and the movement loss L_{stock} (a binary cross-entropy) operate on clearly different scales. The balancing weight λ therefore mediates the trade-off empirically rather than by matching the raw loss scales.

Dataset	$L_{\text{time}}^{\text{final}}$	$L_{\text{stock}}^{\text{final}}$
CIKM24	2.14	0.59
AAAI24	1.70	0.47
SP500	1.86	0.47
CSI300	0.97	0.66
NI225	1.83	0.51
EURO50	1.82	0.50
