# Supplementary material for: Accurate stock movement prediction via centroid-based randomness smoothing
Source: PLoS One. 2026 Oct 8;21(10):e0345252. doi: 10.1371/journal.pone.0345252 (PMC13649149; doi:10.1371/journal.pone.0345252)
Supplement: Supplementary file 2 [file pone.0345252.s002.pdf]

S2 Table. Per-stock up-day rate distribution over the recent-volatile test window.

The medians lie near 0.50—slightly below it for the Chinese markets (CSI300 and AAAI24), which are mildly down-skewed—thus an accuracy above 0.50 reflects genuine predictive signal rather than an exploitable label imbalance, in agreement with the aggregate base rates reported in the main text.

Dataset	Stocks	Median	IQR (25–75%)	Aggregate
CIKM24	100	0.513	0.501–0.527	0.512
AAAI24	70	0.484	0.460–0.498	0.469
SP500	50	0.524	0.512–0.539	0.523
CSI300	300	0.465	0.438–0.486	0.466
NI225	50	0.505	0.476–0.524	0.508
EURO50	40	0.515	0.484–0.545	0.515
