# Supplementary material for: Accurate stock movement prediction via centroid-based randomness smoothing
Source: PLoS One. 2026 Oct 8;21(10):e0345252. doi: 10.1371/journal.pone.0345252 (PMC13649149; doi:10.1371/journal.pone.0345252)
Supplement: Supplementary file 3 [file pone.0345252.s003.pdf]

**S3 Table. Investment performance under a two-sided transaction cost.** Annualized Sharpe ratio (ASR) and relative maximum drawdown (RMDD, %) for the recent-volatile regime when 6 bps is charged on buys as well as on sells, averaged over five random seeds. The one-way values are in Table 10 of the main text. Equal Weight and Buy-and-Hold are omitted because they are passive and hold their positions, so a turnover-driven cost leaves them essentially unchanged and the comparison is not like for like.

The main-text investment simulation charges a one-way cost of 6 bps on sells. Under the stricter two-sided cost, Craft retains the highest ASR, and the lowest relative maximum drawdown, on four of the six datasets.

| | CIKM24 (US) | | AAAI24 (China) | | S&P500 (US) | |
|---|---|---|---|---|---|---|
| Method | ASR | RMDD | ASR | RMDD | ASR | RMDD |
| LSTM | 0.71 | 16.9 | 0.54 | 22.2 | 0.69 | 7.7 |
| ALSTM | 0.63 | 19.0 | 0.51 | 22.6 | **1.28** | 6.8 |
| PatchTST | −0.07 | 25.0 | −0.08 | 33.2 | 0.32 | 8.3 |
| Informer | −2.46 | 29.5 | 0.43 | 23.7 | 0.23 | 8.6 |
| Autoformer | 0.64 | 18.3 | 0.66 | 19.7 | 0.84 | 7.8 |
| FEDformer | 0.73 | 18.4 | 0.57 | 21.6 | 0.61 | 9.1 |
| MASTER | 0.22 | 23.8 | 0.54 | 21.1 | 0.75 | 7.4 |
| MATCC | −0.20 | 16.6 | 0.73 | 18.8 | −0.27 | 6.0 |
| **Craft** | **1.15** | 12.4 | **1.04** | 16.8 | 1.23 | 6.2 |

| | CSI300 (China) | | NIKKEI225 (Japan) | | EURO50 (Europe) | |
|---|---|---|---|---|---|---|
| Method | ASR | RMDD | ASR | RMDD | ASR | RMDD |
| LSTM | −1.61 | 20.6 | 0.06 | 14.0 | −0.19 | 7.6 |
| ALSTM | −1.62 | 21.2 | −0.11 | 17.4 | 0.19 | 8.1 |
| PatchTST | −0.05 | 12.3 | −0.97 | 13.4 | −0.53 | 7.7 |
| Informer | 0.06 | 13.4 | −0.54 | 17.2 | −0.30 | 9.2 |
| Autoformer | 0.11 | 12.9 | 0.16 | 14.0 | −0.05 | 8.6 |
| FEDformer | 0.01 | 12.8 | **0.19** | 13.9 | 0.04 | 8.3 |
| MASTER | −2.09 | 15.4 | −0.56 | 17.5 | 0.11 | 8.3 |
| MATCC | 0.15 | 11.8 | −0.16 | 9.7 | 0.18 | 8.1 |
| **Craft** | **1.08** | 8.3 | −0.35 | 11.2 | **0.73** | 6.6 |
